# Effect of Heteroatom Doping on Electrochemical Properties of Olivine LiFePO_4_ Cathodes for High-Performance Lithium-Ion Batteries

**DOI:** 10.3390/ma17061299

**Published:** 2024-03-11

**Authors:** Xiukun Jiang, Yan Xin, Bijiao He, Fang Zhang, Huajun Tian

**Affiliations:** Key Laboratory of Power Station Energy Transfer Conversion and System of Ministry of Education and School of Energy Power and Mechanical Engineering, North China Electric Power University, Beijing 102206, China

**Keywords:** LiFePO_4_, lithium–ion batteries, cathode, heteroatom doping, electrochemical performance

## Abstract

Lithium iron phosphate (LiFePO_4_, LFP), an olivine–type cathode material, represents a highly suitable cathode option for lithium–ion batteries that is widely applied in electric vehicles and renewable energy storage systems. This work employed the ball milling technique to synthesize LiFePO_4_/carbon (LFP/C) composites and investigated the effects of various doping elements, including F, Mn, Nb, and Mg, on the electrochemical behavior of LFP/C composite cathodes. Our comprehensive work indicates that optimized F doping could improve the discharge capacity of the LFP/C composites at high rates, achieving 113.7 mAh g^−1^ at 10 C. Rational Nb doping boosted the cycling stability and improved the capacity retention rate (above 96.1% after 100 cycles at 0.2 C). The designed Mn doping escalated the discharge capacity of the LFP/C composite under a low temperature of −15 °C (101.2 mAh g^−1^ at 0.2 C). By optimizing the doping elements and levels, the role of doping as a modification method on the diverse properties of LFP/C cathode materials was effectively explored.

## 1. Introduction

In recent decades, traditional energy consumption, notably that of coal and oil, has escalated, necessitating the exploration of developing renewable energy technologies to support energy consumption in a sustainable modern society. Based on the development of the sustainable energy transition, electrochemical energy storage (EES) technology has emerged [1,2,3]. EES systems are regarded as efficient systems which can store energy through chemical reactions to convert chemical energy into electric energy. According to the different reaction mechanisms, it can contain different energy storage technologies such as lead–acid batteries, sodium–sulfur batteries, liquid metal batteries, lithium–ion batteries (LIBs) [2], etc. Simultaneously, advanced EES systems featuring a high energy density, long–term cycle life, and superior energy conversion efficiency can effectively satisfy the demand of the peak–frequency regulation of power systems [2,3].

As an emerging energy storage device, LIBs have been widely applied due to their safety, high energy, long life, and good thermal stability [4,5]. In particular, the electrochemical properties of LIBs, such as their cycling lifespan, energy density, and rate performance, largely depend on the electrochemical properties of the selected cathode materials [6]. Therefore, the exploration of high–performance LIB cathode materials is one of the key directions to promote the further applications of LIBs in EES systems [7]. Currently, cathode materials for LIBs, including LiCoO_2_ (LCO), LiMn_2_O_4_ (LMO), LiNi*_x_*Co*_y_*Mn_1−*x*−*y*_O_2_ (NCM), LiNi*_x_*Co*_y_*Al_1−*x*−*y*_O_2_ (NCA), and LiFePO_4_ (LFP), are extensively utilized. Each of these cathode materials has its own advantages and disadvantages in terms of energy density, cost, stability, lifetime, and safety. As one of the leading candidates for LIB cathode materials, LFP cathodes are widely used in various electrical appliances and electric vehicles due to their reasonable specific capacity (170 mAh g^−1^) [8], excellent cycling stability, and low cost. However, their lower Li^+^ diffusion coefficient (~10^−16^–10^−14^ cm^2^ s^−1^) and lower electrical conductivity (<10^−9^ S cm^−1^) [9,10] limit the further development of LFP cathodes. Therefore, diversified strategies have been undertaken to enhance the electrochemical performance of LFP cathodes, mainly including grain size tuning [11], carbon coating [12], and heteroatom doping [11,13]. For example, reducing the size of LFP particles aids in shortening the Li^+^ diffusion distance, thereby enhancing the rate performance and cycling stability. Carbon coatings can improve the electrical conductivity of cathode materials and further optimize the electrochemical performance of LFP cathodes. Meanwhile, carbon species can impede the growth of LFP grains during heat treatment, and they can be used as reduction agents to avoid the oxidation of Fe^2+^ to Fe^3+^. In addition, the problem of the low electrical conductivity of LFP could be partially solved by heteroatom doping. Yet, the doping mechanism is complex and requires further study.

Carbon coatings can fundamentally be categorized into in situ and ex situ coatings [14,15,16]. The in situ coating strategy requires the addition of an organic carbon source during the preparation of cathode materials. The organic carbon source can be decomposed to form carbon monomers, which are then attached to the surface of the LFP particles during the heat treatment process. Studies have suggested that in situ coating is more effective than ex situ coating with the direct addition of carbon materials [16,17,18,19]. The carbon sources used for carbon coatings are mainly organic carbon sources, including glucose, sucrose, citric acid, and cotinine black. These carbon sources have a strong affinity for the LFP surface and can be thermally decomposed during heat treatment. Due to their film–forming properties, a carbon coating is formed to encapsulate the LFP surface uniformly, thus promoting electrical conductivity [20,21]. Generally speaking, a carbon coating on a cathode material mainly enhances the surface conductivity, yet it has no significant impact on the internal material electrical conductivity due to the inherent structure. It is worth noting that the lattice structure can be altered by doping ions during the preparation of cathode materials. This may help to increase the intrinsic conductivity, Li^+^ diffusion coefficient, and charge–discharge voltage plateau of the materials, thereby improving the rate performance, cycling performance, and low–temperature electrochemical performance of the batteries. For doping modification, most common single-element doping techniques have been reported, and even synergistic modification by the co–doping of multiple elements has been applied. Due to the complexity of the doping mechanism, many types of cation doping, such as with Mg^2+^, Nb^3+^, Co^2+^, Ti^4+^, and V^5+^, and anion doping, such as with F^−^, Cl^−^, etc., have not been fully understood [22,23,24,25,26,27].

In this work, we used FePO_4_ (FP) as the precursor material and adopted the ball milling method to prepare LFP/C composites. We investigated the effects of different doping elements (F, Mn, Nb, and Mg) and doping amounts on the electrochemical properties of LFP/C composite cathodes. Specifically, our work presents a method for creating high–performance olivine–type cathode materials that are specifically suitable for LIBs under low–temperatrure operation conditions (−15 °C).

## 2. Experimental Section

### 2.1. Synthesis and Doping of LFP/C

As a control sample, an LFP/C cathode material was fabricated via the sol−gel method. We added FePO_4_ (Macklin, Shanghai, China, 99%) and CH_3_COOLi·H_2_O (Aladdin, Shanghai, China, 99%) into 20 mL of deionized water (Aladdin, Shanghai, China, AR) at a molar mass ratio of 1:1.05, followed by the addition of 15 wt.% ethylene glycol (Macklin, Shanghai, China, 99%) as well as 20 wt.% citric acid (C_6_H_8_O_7_, Macklin, Shanghai, China, 99%). Ethylene glycol serves as a complexing agent, increasing solution viscosity for sol–gel formation. Citric acid acts as both a carbon source and a complexing agent. The solution was stirred in a water bath at a temperature of 80 °C until a sol was formed. The sol was dried overnight at 80 °C in a vacuum oven before being fully ground in a mortar for 40 min and sieved (200 mesh). Then, the sieved powder was calcined in an OTF–1200X tube furnace (Hefei KeJing, Hefei, China) at 300 °C for 3 h and 700 °C for 7 h in an Ar atmosphere. Figure S1 shows the charge–discharge curve of the first cycle of the LFP/C prepared by the sol–gel method and its cycling performance. It can be seen that the cycling performance of the sample was poor, and the capacity retention rate was only 60% after 100 cycles at 0.2 C.

**Figure 1:**
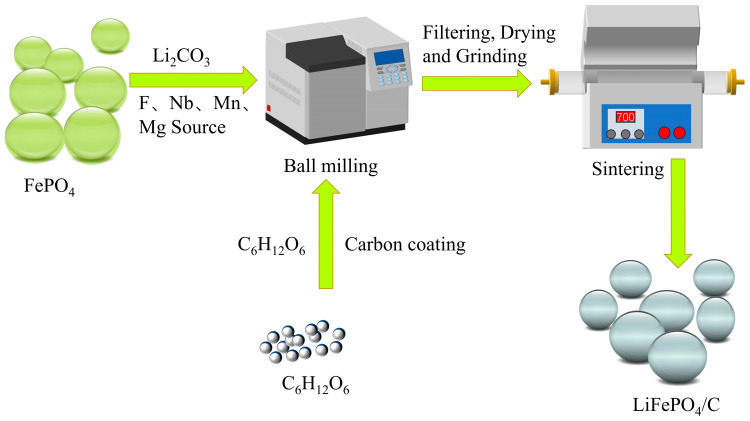
Simple schematic preparation process of LFP/C cathode materials using a carbothermal reduction combined with a synergistic strategy of bulk–phase doping.

The studied LFP/C cathode materials were synthesized using the ball milling method coupled with a heat treatment. Firstly, the precursors FePO_4_ and Li_2_CO_3_ (calculated as 5% excess, Macklin, Shanghai, China, 99%); various dopant sources, including LiF (Macklin, Shanghai, China, 99%), Mn (CH_3_COO)_2_·4H_2_O (Macklin, Shanghai, China, 99%), and C_10_H_5_NbO_20_·*x*H_2_O (Aladdin, Shanghai, China, 99%); and the carbon source of glucose (Macklin, Shanghai, China, 99%) were placed in a ball milling jar, along with an appropriate amount of ethanol(Lanyi, Beijing, China, 99.8%) as a dispersant. The mixture was ground at a speed of 400 r/min for 12 h, filtered, and dried overnight at 80 °C. The dried filter cake was thoroughly ground for 40 min in a mortar and sieved (200 mesh). This powder was transferred to the OTF–1200X tube furnace for initial calcination at 300 °C for 3 h in an Ar atmosphere, followed by a secondary heat treatment at 700 °C for 7 h. The heating/cooling rate of the calcination process was 5 °C/min, and the gas flow rate was maintained at 0.5 L/min. The resulting modified materials were labeled as LFP/C and LFP/C–X*_n_*, respectively (X is the dopant element, and *n* is the ratio of molar fraction of the doping element, X = F, Mn, Nb, and Mg). The preparation process of the LFP/C cathode materials is illustrated in Figure 1.

To assess the electrochemical performance of the LFP/C and LFP/C–X*_n_*, the preparation of cathode disks was carried out. Sintered LFP/C active material, conductive carbon black (Super P, conductive agent), and polyvinylidene fluoride (PVDF, binder) were weighed at a ratio of 8:1:1. The weighed PVDF was added to a stirring bottle prior to the introduction of an appropriate amount of N–methyl–2–pyrrolidone (NMP). The solution underwent magnetic stirring until the PVDF was completely dissolved in the NMP. Meanwhile, the weighed active material and Super P were homogenized in a mortar to obtain a uniform mixture. The well–mixed material was added to the stirring bottle and continued to be stirred for 6 h to achieve a homogeneous slurry. Subsequently, the slurry was uniformly deposited onto Al foil using an applicator and dried overnight at 120 °C, and then punched and pressed into 12 mm diameter cathode disks.

### 2.2. Material Characterization

The crystal structures of the synthesized samples were elucidated via X–ray diffraction (XRD, PANalytical X´Pert3 Powder, Alemlo, The Netherlands). A diffractometer, utilizing Cu–Kα radiation (λ = 0.15418 nm) at 36 kV and 30 mA and scanning at 2° min^−1^, was utilized to record XRD patterns from 2θ = 10° to 80°. Scanning electron microscopy (SEM, Hitachi S–4800, Tokyo, Japan) was employed for surface morphology analysis. Simultaneously, an energy-dispersive spectrometer (EDS, Oxford EMAX, Oxford, UK) was employed for element mapping to verify homogeneous doping. Fourier-transform infrared (FT-IR, Shimadzu IR Affinity-1S spectrometer, Shimadzu, Kyoto, Japan) spectra in the range of 2000–400 cm^−1^ were recorded by a Shimadzu IRAffinity-1s spectrometer. Surface compositions and included elements were characterized using X-ray photoelectron spectroscopy (XPS, EscaLab 250Xi, Perkin Elmer, Waltham, MA, USA). The X-ray source was an aluminum (Al) and magnesium (Mg) thin-film dual-anode target with a spot size of 650 μm. C 1s (284.8 eV) was selected as the calibration value. A thermogravimetric analyzer (TGA, TGDTA7300, Tokyo, Japan) was utilized to study the mass variation in the sample versus temperature shift. In this experiment, the sintered LFP was thermogravimetrically analyzed to examine the carbon content under the following conditions: air atmosphere, temperatures between 20 °C and 800 °C, heating rate of 10 °C min^−1^.

### 2.3. Electrochemical Characterization

All coin–type cells (CR2032) were assembled in an argon–filled glovebox, utilizing the prepared cathode disk as the cathode, the Celgard 2500 apparatus as a separator, and Li foil for counter electrodes. As an electrolyte, 1 mol L^−1^ LiPF_6_ dissolved in organic solvents of dimethyl carbonate/ethylene carbonate/ethyl methyl carbonate (DMC: EC: EMC = 1:1:1 by volume) and 1 vol.% vinylidene carbonate (VC) was used.

Galvanostatic charge–discharge tests between 2.5 and 4.2 V at 25 °C were performed by a Land battery tester (CT3002A), initially at 0.2 C (1 C = 170 mA g^−1^) for the first three cycles, followed by additional charge–discharge assessments at 0.2 C, 0.5 C, 1.0 C, 2.0 C, 5.0 C, and 10.0 C. In addition, the low–temperature electrochemical performance was evaluated at −15 °C in a high–low–temperature test chamber (BPH−060B, Shanghai Yiheng, Shanghai, China).

Electrochemical Impedance Spectroscopy (EIS) and Cyclic Voltammetry (CV) were conducted utilizing an electrochemical workstation (CHI760E, Chenhua, Shanghai, China). CV tests were operated between 2.5 and 4.2 V at 0.1–0.5 mV s^−1^. EIS tests were carried out by sweeping frequencies from 100 kHz to 0.01 Hz at an amplitude of 5 mV.

## 3. Results and Discussion

**Figure 2:**
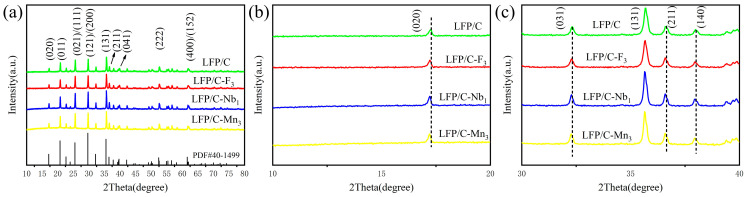
(**a**) XRD patterns of LFP/C and LFP/C modified by doping with different elements, (**b**) the zoomed–in patterns in the 2θ range of 10–20°, and (**c**) the zoomed–in patterns in the 2θ range of 30–40°.

**Figure 3:**
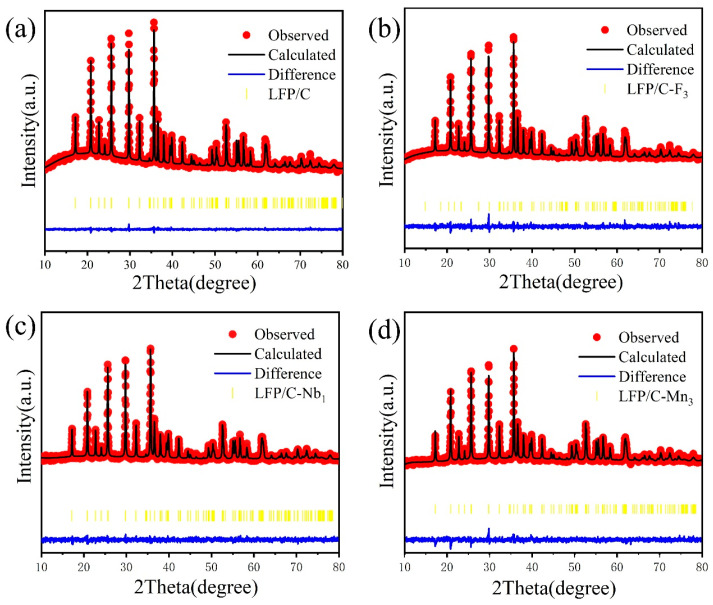
Rietveld refinements of the XRD patterns of (**a**) LFP/C, (**b**) LFP/C–F_3_, (**c**) LFP/C–Nb_1_, and (**d**) LFP/C–Mn_3_ samples.

Figure 2, Figure 3, and Figure S2 show the XRD patterns of the prepared LFP/C and LFP/C–X*_n_* materials with their refinement results and associated structural details (PDF#40–1499). All the samples have similar XRD diffraction peaks, all of which can be indexed to an orthorhombic crystal system (the space group Pnma). No peaks related to dopant elements appeared, as only trace amounts of dopant elements (F, Mn, Nb, and Mg) were contained in the doped LFP samples.

Figure 2b,c present the local amplification of (020), (031), (211), and (140) diffraction peaks. When doped with other elements, the diffraction peaks shifted to lower 2θ degrees. The ionic radii of Mn^2+^, Nb^5+^, Mg^2+^, and F^−^ are 0.83 Å, 0.64 Å, 0.72 Å, and 1.33 Å, respectively, compared to O^2−^ (1.4 Å) and Fe^2+^ (0.74 Å). Therefore, Nb^5+^, Mg^2+^, and Mn^2+^ can substitute Fe^2+^ for diffusion into the octahedral interstices on the LFP surface lattice, while Fˉ can easily replace O^2−^ in the LFP lattice [28,29]. Furthermore, Tables S1 and S2 exhibit the lattice parameters obtained by structural refinement in Figure 3. As shown in Figure 2b,c and Table S1, the variation patterns of lattice parameters *a*, *b*, and *c* are consistent with the shifts of the (020), (031), (211), and (140) diffraction peaks. The lattice parameter decreased, while the diffraction peaks were all shifted towards the left direction. According to the refinement results in Tables S1 and S2, the lattice parameters *a*, *b*, and *c* of the doped LFP/C are less than those of the undoped LFP/C, suggesting that elemental doping affects the crystal structure of LFP/C by influencing the lattice parameters. Inside the LFP lattice, Li^+^ migration is one–dimensional, and reducing the lattice parameter *b* can shorten the Li^+^ diffusion pathway and increase the Li^+^ diffusion coefficient, thus improving the electrochemical properties of the samples [13].

**Figure 4:**
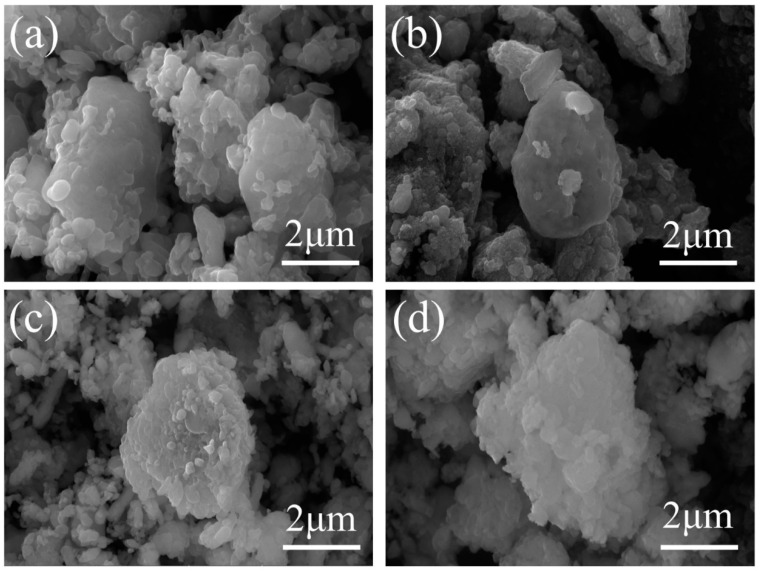
SEM images of (**a**) LFP/C, (**b**) LFP/C–Nb_1_, (**c**) LFP/C–Mn_3_, and (**d**) LFP/C–F_3_ particles.

In addition, SEM and EDS elemental mapping were performed to observe the morphologies and compositions of the LFP/C and doped samples. SEM images of the precursor FePO_4_, LFP/C, and doped modified LFP/C–X*_n_* are shown in Figure 4 and Figure S3. It can be observed that there was no significant change in the morphology of the particles of the doped modified sample compared to the undoped sample. The size of the cathode material particles ranged from 2 to 5 μm. The secondary particles were aggregated from primary particles [30,31]. Figures S4–S7 illustrate the elemental mapping in the LFP/C and LFP/C–X*_n_*. In the EDS spectra, the distribution of doping elements F, Nb, Mn, and matrix elements Fe, P, and O almost coincide uniformly, indicating that the doped elements were successfully doped into the bulk phase of the LFP matrix, while the C elements are mainly distributed on the surface of the LFP particles, indicating that the carbon film formed by the carbon coating was uniformly distributed around the surface of the LFP cathode.

To further confirm that the carbon in the materials was produced by the pyrolysis of glucose, FT–IR was performed on glucose, LFP, LFP/C and doped cathode materials. The FT–IR spectra shown in Figure S8 indicate that the thermal decomposition of glucose occurred during the heat treatment, resulting in the formation of a uniform and dense carbon coating layer around the LFP particles. This layer could largely enhance the electrical conductivity of the LFP/C cathode materials [32,33]. From the FT–IR spectra, the carbon–coated LFP/C showed blue–shifted absorption bands in the ranges of 1200–900 cm^−1^ and 700–400 cm^−1^ with additional absorption peaks of ~1141 cm^−1^ as compared to that of the LFP without carbon coating, which further proves that glucose interacts with LFP during heat treatment [34,35].

TGA tests were performed on the cathode materials to roughly calculate the carbon contents of the materials. The TGA plots in Figure S9 reveal a small degree of sample weight loss below 300 °C, which is attributed to crystallization water loss. Beyond 300 °C, LFP/C began to be oxidized with the oxidation products Fe_2_O_3_ and Li_3_Fe_2_(PO_4_)_3_. At this point, the carbon–coated LFP undergoes the following two reactions [36]:

$${LiFePO}_{4}+\frac{1}{4}O_{2}=\frac{1}{3}{Li}_{3}{Fe}_{2}{\left({PO}_{4}\right)}_{3}+\frac{1}{6}{Fe}_{2}O_{3}$$

$${LiFePO}_{4}+xC+\frac{1}{4}O_{2}+xO_{2}=\frac{1}{3}{Li}_{3}{Fe}_{2}{\left({PO}_{4}\right)}_{3}+\frac{1}{6}{Fe}_{2}O_{3}+x{CO}_{2}$$

Based on these two reactions, the theoretical weight gain of pure LFP is 5.07 wt.%. According to the measured TG curves shown in Figure S9, the actual weight gain of the LFP/C–F_3_, LFP/C–Nb_1_, and LFP/C–Mn_3_ samples were 3.07, 3.16, and 2.6 wt.%, respectively. The percentage of carbon (C_wt_%) can be calculated using the following equation [36]:

(1) $$C_{\mathrm{wt}}\%=1-\frac{1+a\%}{1+b\%}$$

In Equation (1), *a* is the percentage of actual weight increase for the samples and *b* is the percentage of theoretical weight increase for the pure LFP. The carbon contents in LFP/C–F_3_, LFP/C–Nb_1_, and LFP/C–Mn_3_ were calculated as 1.90, 1.82, and 2.35 wt.%, respectively [37]. Figure S10 shows the charge–discharge curves of LFP/C samples with varying glucose contents. The discharge capacity of LFP/C increased remarkably with 5% glucose content but gradually diminished with the glucose content further increasing to 20%. In conjunction with Figure S9, only a limited amount of glucose interacts with the LFP surface as the carbon content increases, while partially pyrolyzed carbon produces an excessively thick carbon layer, which leads to negative effects such as higher battery resistance and lower reversible capacity [12,38]. In the LFP/C–X*_n_* preparation process, the carbon content was optimized to be 5%.

In order to further prove that the structural changes from the XRD results were caused by the doping of elements, the elemental compositions and chemical bonding states of LFP/C, LFP/C–F_3_, and LFP/C–Nb_1_ were elucidated using XPS, as illustrated in Figure S11. The full spectrum is presented in Figure S11h. The XPS spectra of all samples show typical peaks for C 1s, P 2p, O 1s, Li 1s, and Fe 2p. Figure S11f,g confirm the presence of Nb 3d_5/2_ and Nb 3d_3/2_ at the 206.64 and 209.39 eV peaks [39,40] and F 1s at the 691.85 eV peak in LFP/C–Nb_1_ and LFP/C–F_3_, respectively, signifying the successful doping of F and Nb elements in the LFP/C cathode material.

**Figure 5:**
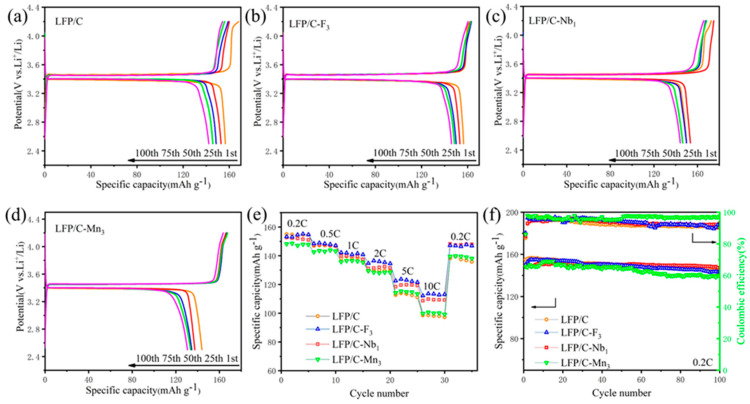
Galvanostatic charge–discharge curves of (**a**) LFP/C, (**b**) LFP/C–F_3_, (**c**) LFP/C–Nb_1_, and (**d**) LFP/C–Mn_3_ samples at different cycles. (**e**) Rate performance of LFP/C and doped modified LFP/C cathodes between 2.5 and 4.2 V (25 °C) and (**f**) cycling performance at 0.2 C.

The electrochemical performances of the LFP/C and doped samples were tested using a variety of test methods and treatments. Figure S12 demonstrates the charge–discharge curves at 0.2 C in the first cycle for the LFP/C and LFP/C–X*_n_* cathode materials. These data indicate that LFP/C, LFP/C–F_3_, LFP/C–Nb_1_, and LFP/C–Mn_3_ have discharge capacities of 156.5 mAh g^−1^, 153.3 mAh g^−1^, 152.5 mAh g^−1^, and 148.1 mAh g^−1^, respectively. Figure S13 presents the irreversible capacity difference between the first lap charge capacity and discharge capacity for each sample; the irreversible capacity of the LFP/C, LFP/C–Mn_3_, LFP/C–Nb_1_, and LFP/C–F_3_ cathodes is 21.9 mAh g^−1^, 19.5 mAh g^−1^, 18 mAh g^−1^, 16 mAh g^−1^, respectively. This figure allows for a more visual comparison of initial Coulombic efficiency. The charge–discharge profiles of LFP/C and LFP/C–X*_n_* at 0.2 C for the 1st, 25th, 50th, 75th, and 100th cycles are depicted in Figure 5a–d. The decaying discharge capacities of the cycled LFP/C, LFP/C–F_3_, LFP/C–Nb_1_, and LFP/C–Mn_3_ cathodes are 14.2 mAh g^−1^, 10.4 mAh g^−1^, 6.9 mAh g^−1^, and 13.3 mAh g^−1^ at 0.2 C, respectively. The rate performances of the LFP/C and LFP/C–X*_n_* are illustrated in Figure 5e. As the rate increases, LFP/C–F_3_ exhibits the highest discharge capacity, and the discharge capacity of LFP/C drops below that of LFP/C–Mn_3_ at 5 C and 10 C. Returning from 10 C to 0.2 C, LFP/C–Nb_1_ and LFP/C–F_3_ maintain the highest capacity, which shows their superior electrochemical performance. It could also indicate that the cycling performance of the doped LFP was improved to some extent. Among the cathodes, the cycling performance of LFP/C–Nb_1_ was the best. Figure 5f compares the cycling performance of these samples at 0.2 C, revealing that LFP/C–Nb_1_ exhibits a higher capacity retention rate of 96.1% after 100 cycles, compared to LFP/C–F_3_ (93.3%), LFP/C–Mn_3_ (90.9%), and undoped LFP/C (90.1%). Combined with the XRD refinement results, F^−^ and Nb^5+^ can be effectively doped into the lattice to change the lattice structure, producing defects favorable to the lattice, reducing the internal resistance of the material and the Li^+^ diffusion path, and improving the electrical conductivity of the material, which in turn improves the high rate performance and cycling performance of the materials. The EIS test and CV test of the Li^+^ diffusion coefficient were conducted on the batteries later to verify our speculation.

**Figure 6:**
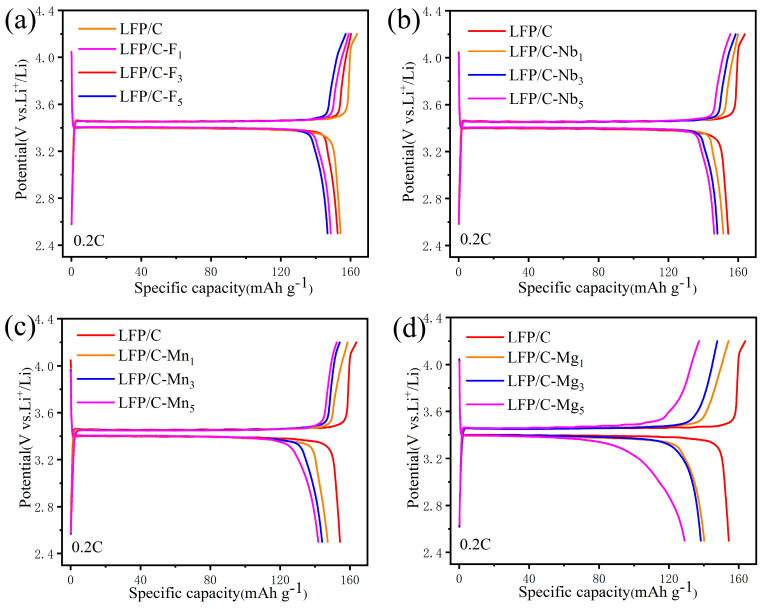
The charge–discharge curves of (**a**) LFP/C–F*_n_*, (**b**) LFP/C–Nb*_n_*, (**c**) LFP/C–Mn*_n_*, and (**d**) LFP/C–Mg*_n_* cathodes at 0.2 C.

To evaluate the electrochemical properties of cathode materials upon cycling, the evolution of the capacity differential curves was investigated. The dQ/dV curves of the samples are shown in Figure S14. During the cycling process, the prepared cathodes underwent a series of phase transitions from the LFP phase to the FP phase, and the reasonable introduction of other elements enhanced the cycling stability of the materials. By observing the dQ/dV curves for each number of cycles and comparing the changes in the curves as the cycle proceeds, the greater the change in the shape of the curves, the more stable the structure of the material is, which also implies a higher reversibility and better cycling stability. By comparison, the LFP/C–Nb_1_ exhibits higher reversibility, corresponding to its superior cycling performance. To visualize the degradation of the specific capacity of samples at different rates, the charge–discharge curves of the samples at rates of 0.2 C–2 C–5 C–10 C were integrated, as shown in Figure S15. From 0.2 C to 10 C, the capacities of LFP/C, LFP/C–F_3_, LFP/C–Nb_1_, and LFP/C–Mn_3_ decrease from 149.9 mAh g^−1^, 155.1 mAh g^−1^, 154.3 mAh g^−1^, and 151.7 mAh g^−1^ to 97.9 mAh g^−1^, 113.7 mAh g^−1^, 108.9 mAh g^−1^, and 100.4 mAh g^−1^, respectively. As the discharging current increases, the LFP/C–F_3_ cathode displays superior rate performance. In order to verify whether the electrochemical performance of the doped LFP at a high rate is the same as that at low rate, the samples were subjected to high–rate long–cycle tests. The cycling performance of the LFP/C and LFP/C–X_n_ cathodes at 2 C and 5 C is illustrated in Figure S16. Notably, the LFP/C–F_3_ cathode outperformed the rest in both discharge specific capacity at 2 C and 5 C–, while the LFP/C–Nb_1_ was able to maintain high capacity retention after 100 cycles. LFP/C–Mn_3_ exhibited enhanced cycling performance compared to LFP/C. Figure 6 shows the charge–discharge curves of LFP after doping with different contents of each element, which shows the optimum doping content of each element. Figure 6 demonstrates that superior electrochemical performance was achieved by LFP/C–F_3_, LFP/C–Nb_1_, LFP/C–Mn_1_, and LFP/C–Mg_1_ when subjected to F, Nb, Mn, and Mg single–element doping, respectively. The Mg doping, however, proved ineffective due to its overall inferior doping effect. Concerning Mn doping, LFP/C–Mn_3_ was chosen for further examination due to its superior low–temperature properties compared to LFP/C–Mn_1_, as depicted in Figure S17. All of these data indicate that appropriate F, Nb doping can improve the cycling stability of LFP/C materials without compromising capacity [41,42]. Figure 5e,f, Figure 6, Figures S15 and S16 confirm the superior discharge capacity and cycling stability of the doped LFP/C–F_3_ and LFP/C–Nb_1_ at different rates, attributed to the enhanced interfacial stability between the electrode and the electrolyte [43,44,45].

**Figure 7:**
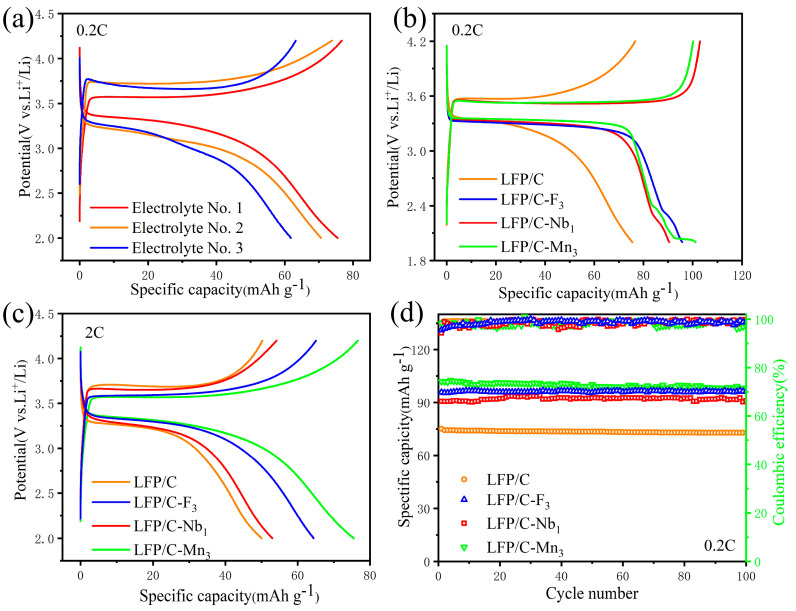
The electrochemical performances of LFP/C and LFP/C–X*_n_* cathodes at low temperature of −15 °C. (**a**) Charge–discharge curves of LFP/C cells assembled with different electrolytes at 0.2 C. Charge–discharge curves of LFP/C, LFP/C–F_3_, LFP/C–Nb_1_, and LFP/C–Mn_3_ cathodes at 0.2 C (**b**) and 2 C (**c**). (**d**) Cycling performance of LFP/C, LFP/C–F_3_, LFP/C–Nb_1_, and LFP/C–Mn_3_ cathodes at 0.2 C.

To investigate the effect of different doping elements on the low–temperature capacity of LFP/C cathode materials, LFP/C cells were assembled using three different electrolytes (electrolyte No. 1, 2, and 3) [46,47,48], which underwent charging and discharging assessments at −15 ℃. Electrolyte No. 1 contained 1M LiPF_6_ in PC: EC: EMC = 1:1:4 Vol%. Electrolyte No. 2 was composed of 1M LiPF_6_ in EC: EMC = 3:7 Vol% + 1%VC, and electrolyte No. 3 consisted of 1M LiPF_6_ in DMC: EC: EMC = 1:1:1 Vol% + 1%VC. Figure 7a depicts the charge–discharge curves of LFP/C batteries at −15 °C at 0.2 C, and the battery assembled with the No. 1 electrolyte demonstrated the highest discharge capacity of 79.4 mAh g^−1^. The battery using the No. 2 and No. 3 electrolytes had reduced capacities of 72.2 mAh g^−1^ and 62.5 mAh g^−1^, respectively. These findings suggest that the optimized electrolytes can significantly boost the capacity of the doped LFP/C cathodes. Therefore, all subsequent low–temperature performance tests were conducted using the No. 1 electrolyte. Figure 7b demonstrates the charge–discharge curves of LFP/C and LFP/C–X*_n_* at 0.2 C, where the highest discharge capacity was achieved by LFP/C–Mn_3_, reaching 102.8 mAh g^−1^ at −15 °C. Figure 7c shows the cycling performances at 2 C, with the LFP/C–Mn_3_, LFP/C–F_3_, and LFP/C–Nb_1_ demonstrating improved discharge capacities of 78.4, 63.7, and 57.1 mAh g^−1^, respectively, over the undoped LFP/C at 50.8 mAh g^−1^. It indicates that the doping of Mn effectively modified the structure of LFP, giving it some of the performance of the LiFe*_x_*Mn_1−*x*_PO_4_ (LFMP), with the low–temperature performance being superior to that of LFP. Meanwhile, the discharge capacity of LFP/C–Nb_1_ decayed faster with increasing current density, which is consistent with its rate properties at room temperature. Figure 7d displays the charge–discharge curves for 100 cycles at 0.2 C (−15 °C), highlighting superior cycling performances at low temperatures, with no significant capacity loss after 100 cycles. In particular, LFP/C–Mn_3_ displayed superior low–temperature discharge capacity and rate performance compared to the undoped LFP/C, LFP/C–F_3_, and LFP/C–Nb_1_, suggesting that rational Mn doping can effectively enhance the low–temperature performance of the LFP/C. This is attributed to the fact that with the addition of an appropriate amount of Mn, the material exhibits some of the partial electrochemical properties of the LFMP. Although the LFMP is poor in electrical conductivity, it has better low–temperature performance than that of LFP, which is also consistent with the above test results.

**Figure 8:**
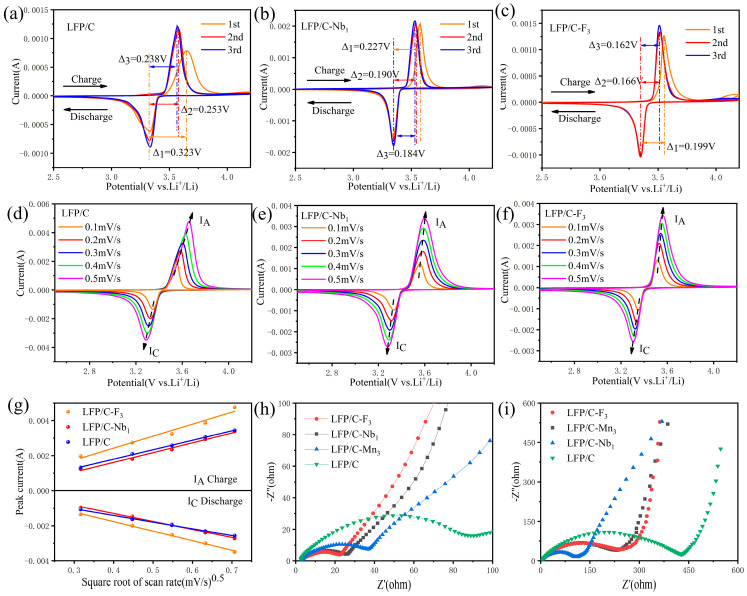
CV curves of (**a**) LFP/C, (**b**) LFP/C–Nb_1_, and (**c**) LFP/C–F_3_ at a scan rate of 0.1 mV s^−1^. CV curves of (**d**) LFP/C, (**e**) LFP/C–Nb_1_, and (**f**) LFP/C–F_3_ at different scan rates, and (**g**) peak current (*i*_p_) versus square root of the scan rate (*v*^1/2^) curves for LFP/C, LFP/C–Nb_1_, and LFP/C–F_3_ samples. Also shown are the Nyquist plots and the fitting data of LFP/C, LFP/C–F_3_, LFP/C–Nb_1_, and LFP/C–Mn_3_ samples at the 3rd (**h**) and the 100th (**i**) cycles.

The constant–current charge–discharge tests, CV measurements, and EIS tests were performed on the samples, and the fitting results were analyzed to support the above conclusions. Figure 8a–c and S18a depict the CV curves of the LFP/C, LFP/C–Nb_1_, LFP/C–F_3_, and LFP/C–Mn_3_ between 2.5 and 4.2 V at a rate of 0.1 mV s^−1^. The data reveal a pair of characteristic peaks that mark Li^+^ extraction and intercalation phase transitions. The potential difference (ΔE), defined as the difference between the peak oxidation voltage and the peak reduction voltage, provides crucial insights into electrode polarization and the reversibility of redox reactions in materials [49]. A lower ΔE value indicates a lower polarization degree of the electrode reaction and faster Li^+^ transfer during cycling, thereby improving stability and reversibility [50]. Therefore, it can be concluded that LFP/C–F_3_ and LFP/C–Nb_1_ possess superior structural stability and phase transition reversibility [51,52], with their ΔE values in the first cycle (0.199 V and 0.227 V, respectively) being lower than both LFP/C–Mn_3_ and the undoped LFP/C (0.253V and 0.323 V, respectively). The subsequent ΔE values at the second and third cycles corroborate this finding.

To assess the effect of doping modification on Li^+^ transport at the surface of the LFP/C electrodes, CV analysis was executed at different scan rates (0.1–0.5 mV s^−1^), as shown in Figure 8d–f and S18b. A linear correlation of the open–square value of the CV scan rate (*v*^1/2^) with the peak current (*i*_p_) yields the slopes of the charging and discharging processes (I_A_ and I_C_), indirectly representing the Li^+^ diffusion coefficient of the samples (*D_Li+_*). For the precise determination of *D_Li+_*, the Randles–Sevcik equation should be used, as shown in Equation (2) [39,53,54].

(2) $$i_{p}=2.69\;\times \;10^{5}n^{3/2}AD_{L{\mathrm{i}}^{+}}^{1∕2}{Cv}^{1/2}$$

Here, *n* denotes the number of electrons involved in redox reactions; *i*_p_ represents the peak current (mA); *A* signifies the electrode geometric area (cm^2^); *D*_Li+_ denotes the Li^+^ diffusion coefficient; *C* relates to the Li^+^ concentration of (mol cm^−3^); and ***v*** corresponds to the potential scan rate (V s^−1^) [39].

The relation between *D_Li+_* and the slope of *i*_p_–*v*^1/2^ suggests a direct proportionality. A larger slope implies increased *D_Li+_*. The slopes of the curves (I_A_ and I_C_) for all samples’ curves were initially calculated through linear fitting, followed by the calculation of *D_Li+_* during charging (*D_OI_*) and discharging (*D_RI_*) by Equation (2), and the results are shown in Table S3 and Figure 8g; they highlight that LFP/C–F_3_ exhibits superior slopes and subsequent *D_RI_* (1.15 × 10^−11^ cm^2^ s^−1^) compared to the undoped LFP/C, LFP/C–Nb_1_, and LFP/C–Mn_3_ cathodes. The fitting results of the EIS spectra can be seen in Figure 8h,i and Table S4. As illustrated in Figure S19, an equivalent circuit model fitted using the EIS data indicates *R_s_*, *R_ct_*, ω1, and CPE1 correspond to ohmic resistance, charge transfer resistance, Warburg impedance, and nonideal capacitance of the surface layer, respectively [55,56]. Table S4 shows that LFP/C–F_3_ displayed the lowest *R*_s_ value of 1.76 Ω after the initial three cycles, rising to 3.381 Ω after 100 cycles. LFP/C–Nb_1_ displayed a rise of *R*_s_ value from 2.509 Ω to 3.326 Ω, and the *R_s_* value of LFP/C–Mn_3_ grew from 2.844 Ω to 4.231 Ω after 100 cycles. After 100 cycles, the *R_ct_* value of LFP/C–Nb_1_ increased slowly from 20.88 Ω to 110.1 Ω, while the LFP/C’s *R_ct_* escalated dramatically from 76.52 Ω to 378.5 Ω. In contrast, the *R_ct_* values for LFP/C–F_3_ and LFP/C–Mn_3_ rose to 199.7 Ω and 206.14 Ω after 100 cycles. The lower the impedance of the cell, the better the Li^+^ diffusion rate and the better the cycling performance of the cell. The impedance fitting results underscore the superior stability of LFP/C–Nb_1_ under prolonged cycling. The determination of the Li^+^ diffusion coefficient (*D*_Li+_) is outlined in Equations (3)–(5) [39,57,58,59], which are provided below.

(3) $$D_{\mathrm{Li}+}=\frac{R^{2}T^{2}}{2A^{2}n^{4}F^{4}\sigma_{w}^{2}c_{0}^{2}}$$

(4) $$Z^{\prime }={\;R}_{s}+R_{ct}+\sigma_{w}\omega^{1∕2}$$

(5) $$c_{0}=\;m/M/d/A$$

In Equations (3) and (4), *A* signifies the cathode surface area (1.1304 cm^−2^), *n* reflects the number of transferred electrons (1), *F* equates to Faraday’s constant (96,485. 34 C mol^−1^), *R* represents the gas constant (8.314 J mol^−1^ K^−1^), *T* is the absolute temperature (298.15 K), and *ω* denotes the Li^+^ concentration, with *σ_ω_* being the characteristic Warburg factor related to Z′ [39] as calculated from Equation (4) by fitting to Figure S20. In Equations (3) and (5), *c_0_* denotes the active substance concentration in mol cm^−3^; *m* stands for the active substance mass on the test electrode in g; *M* symbolizes the relative molecular weight of the material in g mol^−1^; *d* is the electrode coating thickness in cm; and *A* is the previously described electrode surface area.

This analysis affirms that the *D*_Li+_ of LFP/C was 2.36 × 10^−13^ cm^2^ s^−1^ in the third cycle, with this rapidly declining in subsequent cycles. The *D*_Li+_ values for LFP/C–F_3_, LFP/C–Nb_1_, and LFP/C–Mn_3_ were 8.72 × 10^−13^ cm^2^ s^−1^, 7.14 × 10^−13^ cm^2^ s^−1^, and 4.06 × 10^−13^ cm^2^ s^−1^, respectively. After 100 cycles, the *D*_Li+_ of LFP/C decreased to 5.58 × 10^−14^ cm^2^ s^−1^, while the *D*_Li+_ of LFP/C–F_3_, LFP/C–Nb_1_, and LFP/C–Mn_3_ decreased to 2.15 × 10^−13^ cm^2^ s^−1^, 4.59 × 10^−13^ cm^2^ s^−1^, and 1.37 × 10^−13^ cm^2^ s^−1^, respectively. This signifies the effectiveness of F and Nb modification in enhancing ion transfer kinetics, concurring with the electrochemical performance of the batteries.

## 4. Conclusions

A variety of high–performance LFP/C composite cathodes modified by doping different elements (F, Mn, Nb, and Mg) were prepared via ball milling and carbothermal reduction. Comparing the samples after doping with each element, the elements F, Nb, and Mn could improve the performance of LFP in some respects, while Mg doping was less effective. The results indicate that the optimized LFP/C–F_3_ cathode showed an excellent specific capacity at an elevated rate (123.3 mAh g^−1^ at 5 C and 113.7 mAh g^−1^ at 10 C). The designed LFP/C–Nb_1_ cathode presented stable cycling performance (96.1% capacity retention after 100 cycles at 0.2 C), and the selected LFP/C–Mn_3_ cathode exhibited superior low–temperature performance (101.2 mAh g^−1^ at 0.2 C at −15 °C). Meanwhile, in order to give support to the above results, an EIS test and CV test were conducted, and Li^+^ diffusion coefficients were calculated based on the results of both tests. These calculated and fitted results indicated that the F^−^ and Nb^+^ doped LFPs possessed higher Li^+^ diffusion coefficients and lower impedances, attributed to the improved rate and cycling performance. This study provides a strategic pathway to improve the properties of LFP/C cathode materials through doping modification, intensifying the understanding of the doping mechanism in LFP/C cathode materials for LIBs. Future work will be conducted to explore the effect of elemental doping on LFP, including the elements that have been doped (F, Nb, Mn) and those that have not yet been investigated (e.g., Ti, Al, Zr, Ru, etc.). At the same time, we will increase the content of Mn elements in LFP/C−Mn*_n_* transitioning to LFMP, initiating related studies on LFMP.

## Data Availability

Data are contained within the article.

## Supplementary Materials

The following supporting information can be downloaded at: https://www.mdpi.com/article/10.3390/ma17061299/s1. Figure S1. (a)Charge/discharge curves of LFP cathode materials prepared by sol–gel method at first cycle and (b) the related cycling performance of as–prepared LFP cathode at 0.2C. Figure S2. The optimized structures of (a) LiFePO_4_ and (b) FePO_4_. Figure S3. SEM images of the FePO4 precursor particles (a) and the corresponding magnified images (b). Figure S4. The SEM image and the corresponding elemental mapping images for the selected area of LFP/C particle. Figure S5. The SEM image and the corresponding elemental mapping images for the selected area of LFP/C–F_3_ particle. Figure S6. The SEM image and the corresponding elemental mapping images for the selected area of LFP/C–Nb_1_ particle. Figure S7. The SEM image and the corresponding elemental mapping images for the selected area of LFP/C–Mn_3_ particle. Figure S8. FT–IR spectra of C_6_H_12_O_6_, LFP, LFP/C and LFP/C–X*_n_* cathode materials. Figure S9. TGA curves of LFP/C–Nb_1_, LFP/C–F_3_, LFP/C–Mn_3_. Figure S10. Charge–discharge curves of LFP/C samples with different glucose contents. Figure S11. XPS spectra and the fitting results of LFP/C–F_3_, LFP/C–Nb_1_ and LFP/C for cathode materials. (a) C 1s spectra, (b) P 2p spectra, (c) O 1s spectra, (d) Li 1s spectra, (e) Fe 2p spectra, (f) Nb 3d spectra, (g) F 1s spectra and (h) the full spectrum of the as–prepared samples. Figure S12. The first–cycle charge–discharge curves of LFP/C and LFP/C–X*_n_* cathodes. Figure S13. The first charge–discharge curves of the (a) LFP/C, (b) LFP/C–F_3_, (c) LFP/C–Nb_1_, and (d) LFP/C–Mn_3_ cathodes at 0.2 C. Figure S14. The selected dQ/dV curves of (a) LFP/C, (b) LFP/C–F_3_, (c) LFP/C–Nb_1_ and (d) LFP/C–Mn_3_ at 0.2C in different cycles. Figure S15. Charge–discharge curves of (a) LFP/C, (b) LFP/C–F_3_, (c) LFP/C–Nb_1_ and (d) LFP/C–Mn_3_ under different charge/discharge rates. Figure S16. Cycling performance of LFP/C, LFP/C–F_3_, LFP/C–Nb_1_, and LFP/C–Mn_3_ samples at (a) 2 C and (b) 5C in the range of 2.5–4.2 V at 25 °C. Figure S17. The charge–discharge curves of Mn–doped LFP/C cathodes at 0.2 C at low temperature −15 °C. Figure S18. CV curves of (a) LFP/C–Mn_3_ at a scan rate of 0.1 mV s^−1^ and (b) LFP/C–Mn_3_ at various scan rates. Figure S19. Equivalent circuit diagram fitted and used in EIS data. Figure S20. The relationship between low–frequency Z′ and ω^−0.5^ values derived from EIS results of LFP/C, LFP/C–F_3_, LFP/C–Nb_1_ and LFP/C–Mn_3_ samples after (a) 3 cycles and (b)100 cycles. Table S1. Structural parameters obtained from Rietveld refinements of XRD patterns of the LFP/C and dopant–modified cathode materials [27,60]. Table S2. Rietveld refinement results of the LFP cathode materials modified by doping. Table S3. Slope values of fitted *i_p_*–*v*^1/2^ curves and diffusion coefficients of charged (*D_OI_*) and discharged (*D_RI_*) lithium ions of LFP/C, LFP/C–F_3_, LFP/C–Nb_1_, and LFP/C–Mn_3_ cathodes for CV tests at different scan rates. Table S4. Ohmic resistance (*R*_s_) and charge transfer resistance (*R_ct_*) values and calculated Li^+^ diffusion diffusion coefficients (*D*_Li+_) for LFP/C, LFP/C–F_3_, LFP/C–Nb_1_, and LFP/C–Mn_3_ cathodes after different cycles. Table S5. Specific capacity of multiple LFP/C and doped LFP/C samples at different rates. Table S6. Standard deviation of LFP/C and doped samples at different rates.

## Abbreviations

LFP	LiFePO_4_
FP	FePO_4_
LFP/C	Carbon–coated LiFePO_4_
LFP/C–F*_n_*	Carbon–coated LiFePO_4_ doped with *n* at.% F
LFP/C–Nb*_n_*	Carbon–coated LiFePO_4_ doped with *n* at.% Nb
LFP/C–Mn*_n_*	Carbon–coated LiFePO_4_ doped with *n* at.% Mn
LFMP	LiFeMnPO_4_
LFP/C–X*_n_*	Carbon–coated LiFePO_4_ doped with *n* at.% X dopant element
