# Supplementary material for: Effect of Heteroatom Doping on Electrochemical Properties of Olivine LiFePO4 Cathodes for High-Performance Lithium-Ion Batteries
Source: Materials (Basel). 2024 Mar 11;17(6):1299. doi: 10.3390/ma17061299 (PMC10971814; doi:10.3390/ma17061299)
Supplement: Supplementary file 1 [file materials-17-01299-s001.zip › materials-2870473-supplementary.pdf]

Supplementary Information for

Effect of Heteroatom Doping on Electrochemical Properties of Olivine $LiFePO_4$ Cathodes for High-Performance Lithium-Ion Batteries

Xiukun Jiang, Yan Xin, Bijiao He, Fang Zhang, Huajun Tian **

Key Laboratory of Power Station Energy Transfer Conversion and System of Ministry of Education and School of Energy Power and Mechanical Engineering, North China Electric Power University, Beijing, 102206, China

E-mail: xinyan@ncepu.edu.cn, huajun.tian@ncepu.edu.cn

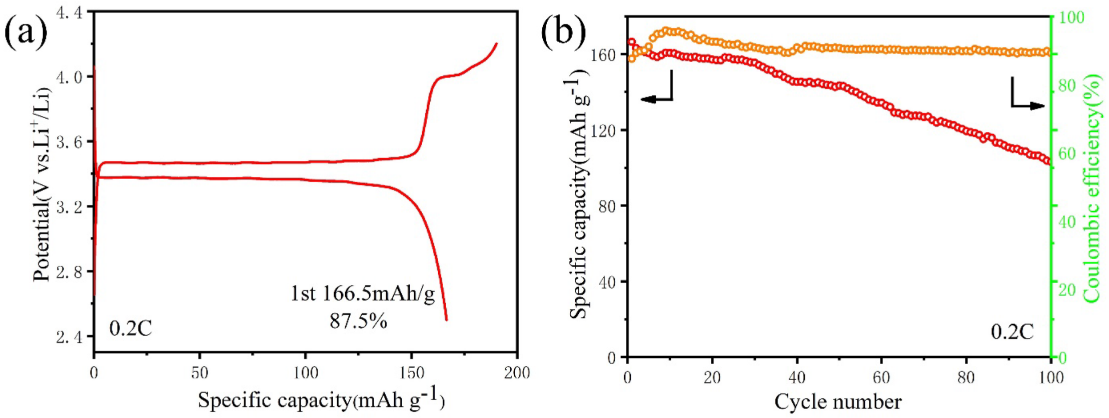

Figure S1. (a)Charge/discharge curves of LFP cathode materials prepared by sol−gel method at first cycle and (b) the related cycling performance of as−prepared LFP cathode at 0.2C.

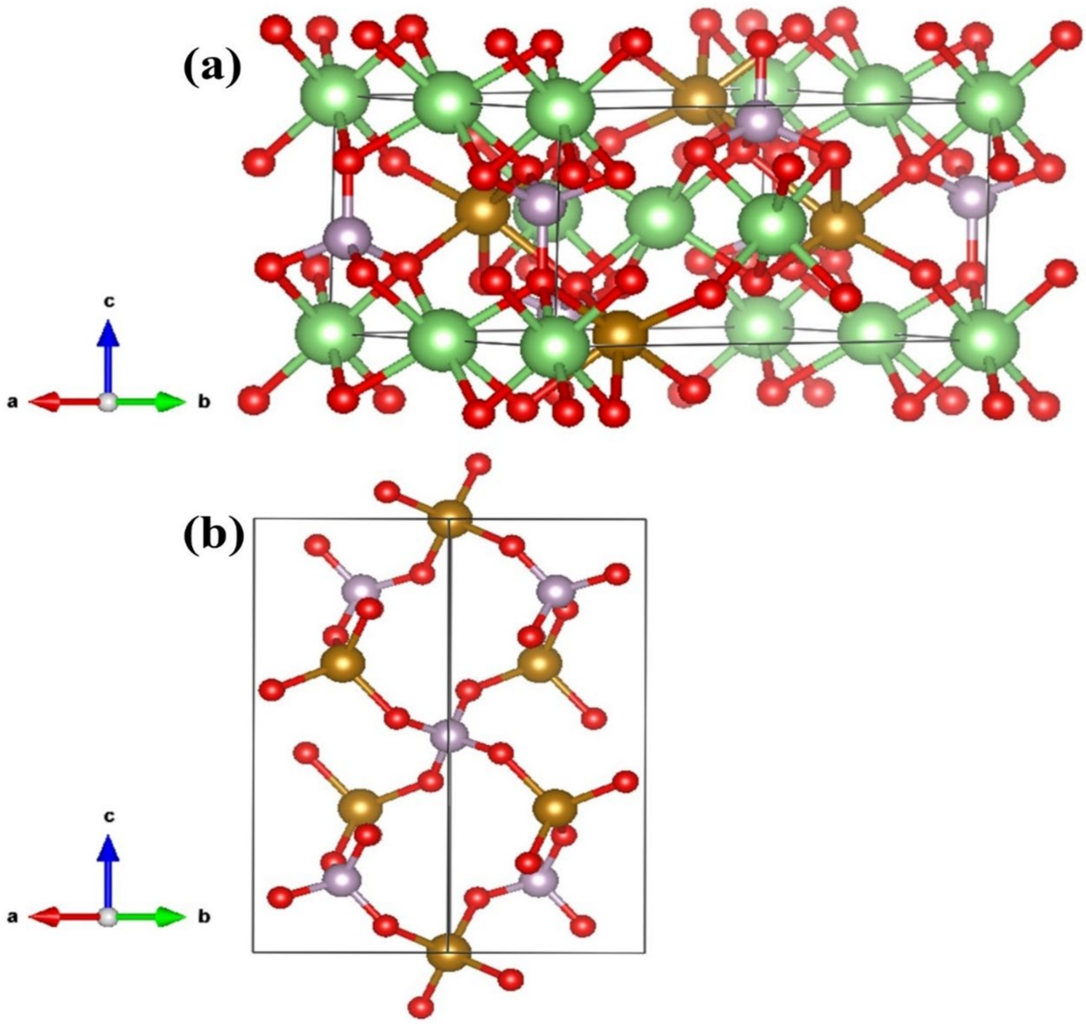

Figure S2. The optimized structures of (a) $LiFePO_4$ and (b) $FePO_4$.

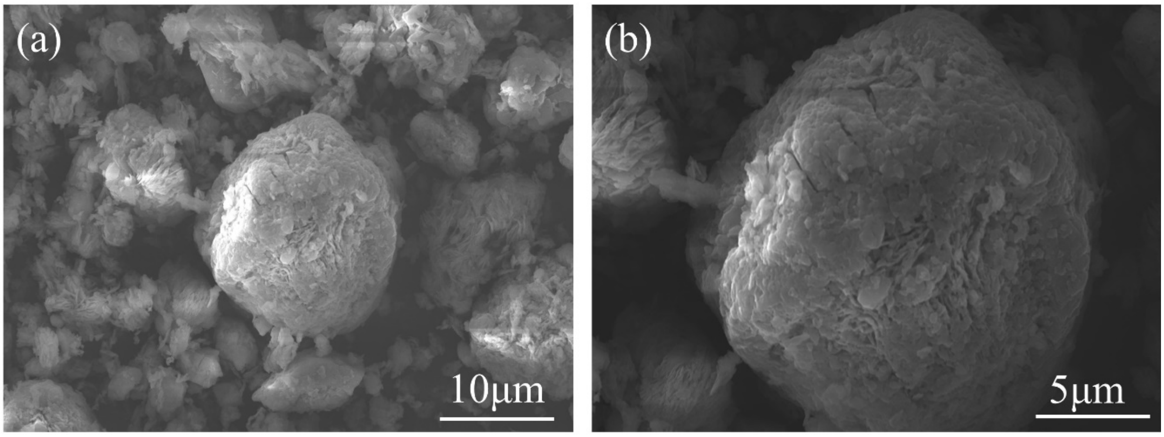

Figure S3. SEM images of the $FePO_4$ precursor particles (a) and the corresponding magnified images (b).

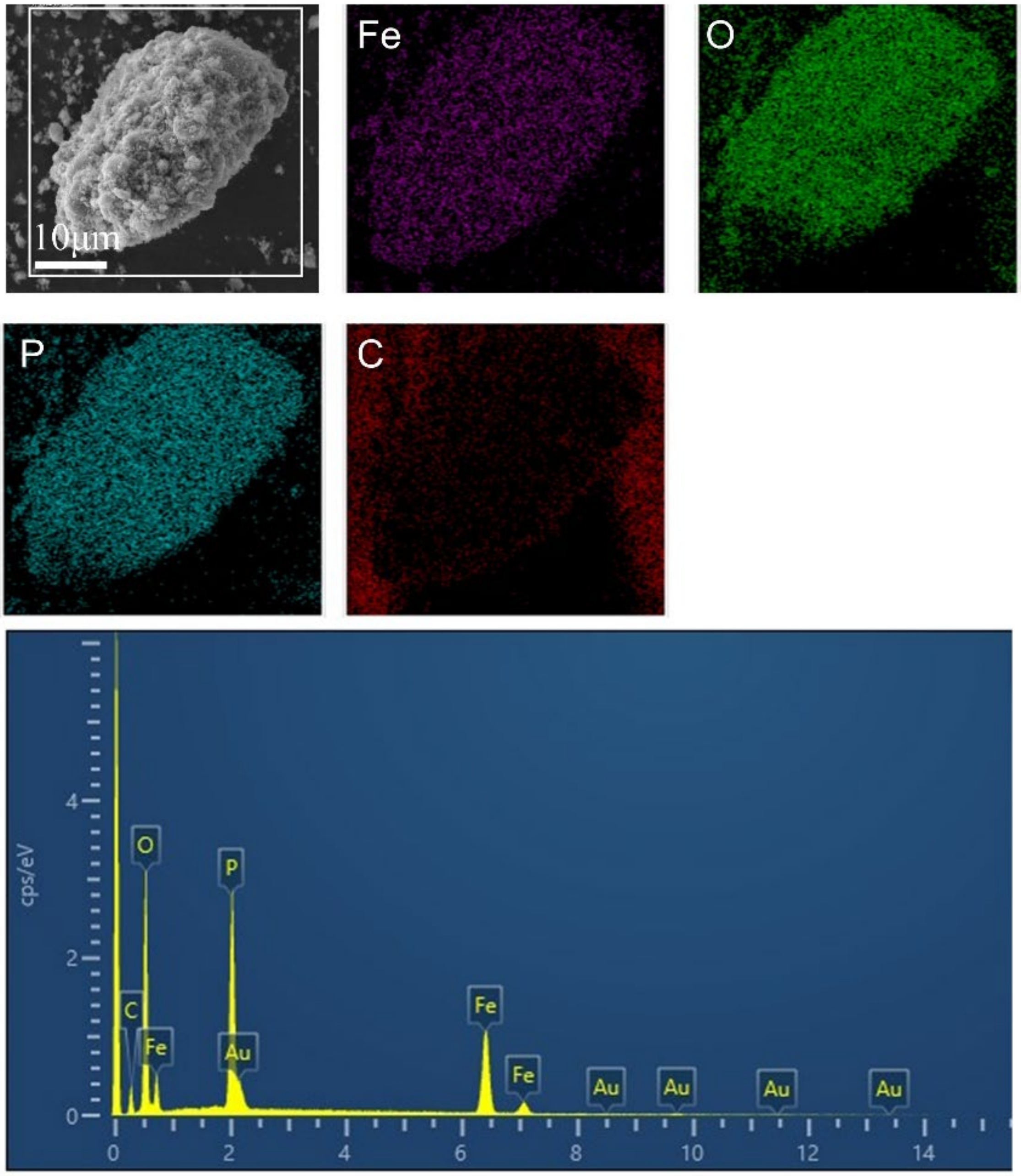

Figure S4. The SEM image and the corresponding elemental mapping images for the selected area of LFP/C particle.

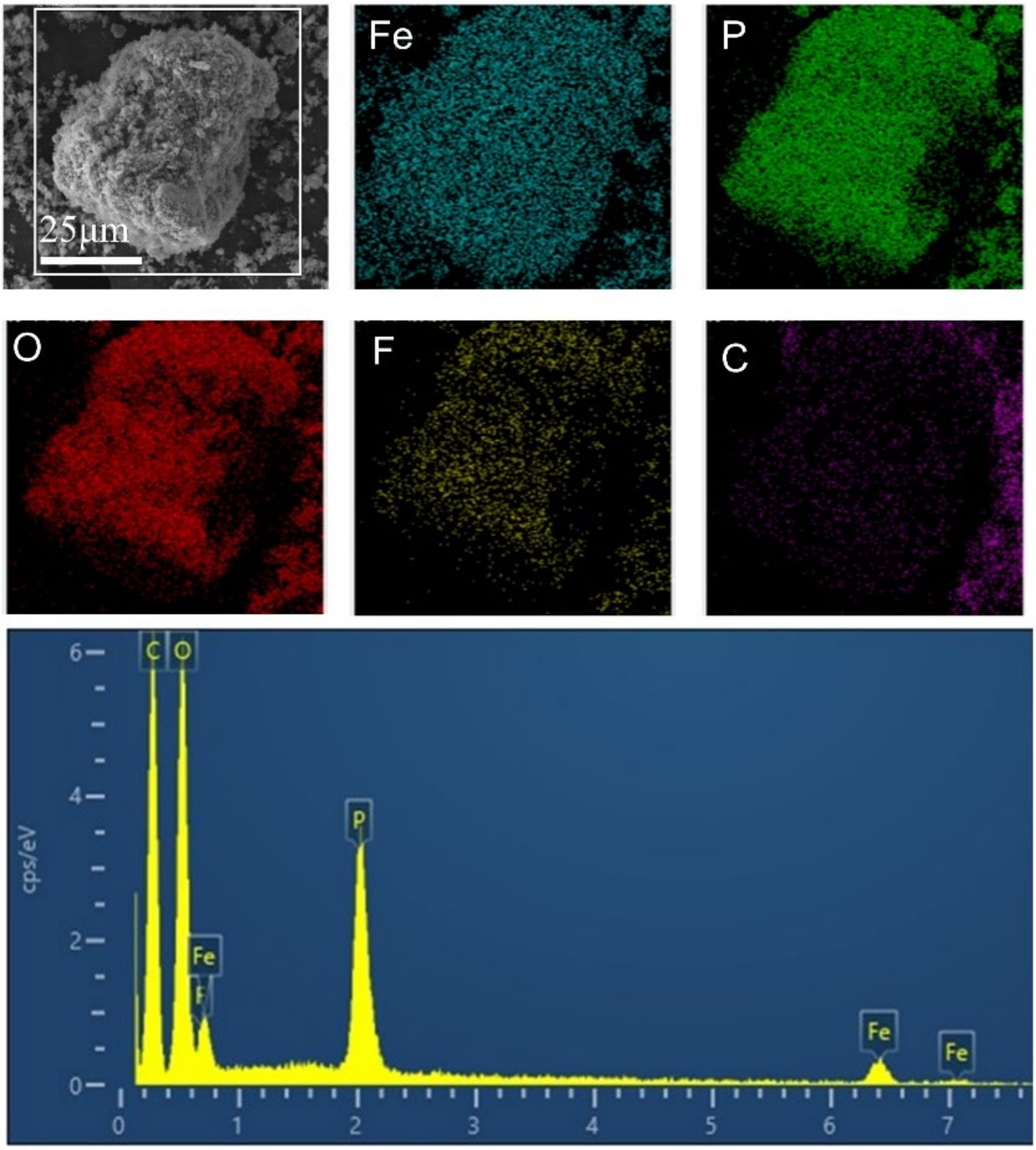

Figure S5. The SEM image and the corresponding elemental mapping images for the selected area of LFP/C−F_3 particle.

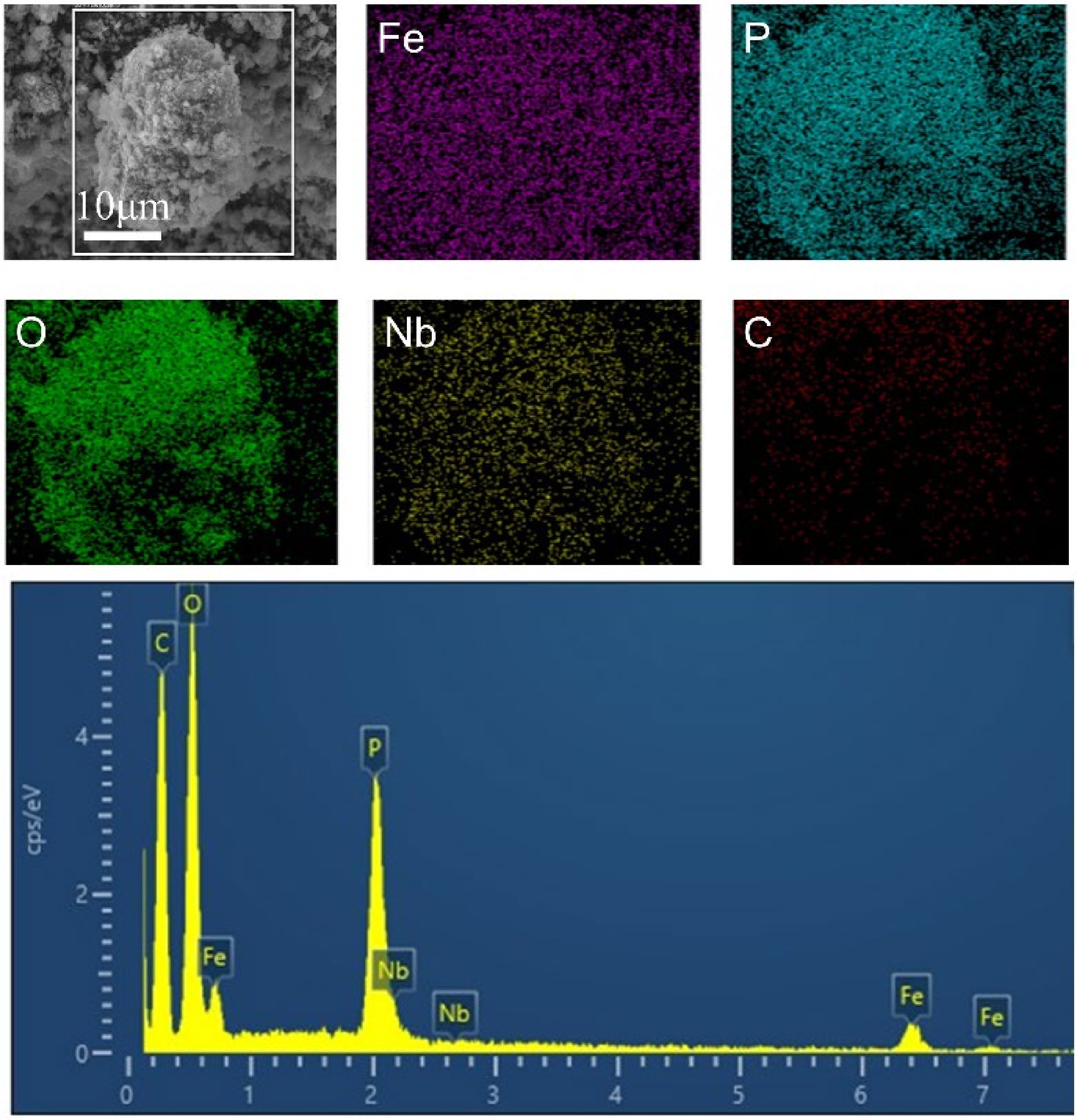

Figure S6. The SEM image and the corresponding elemental mapping images for the selected area of LFP/C−Nb_1 particle.

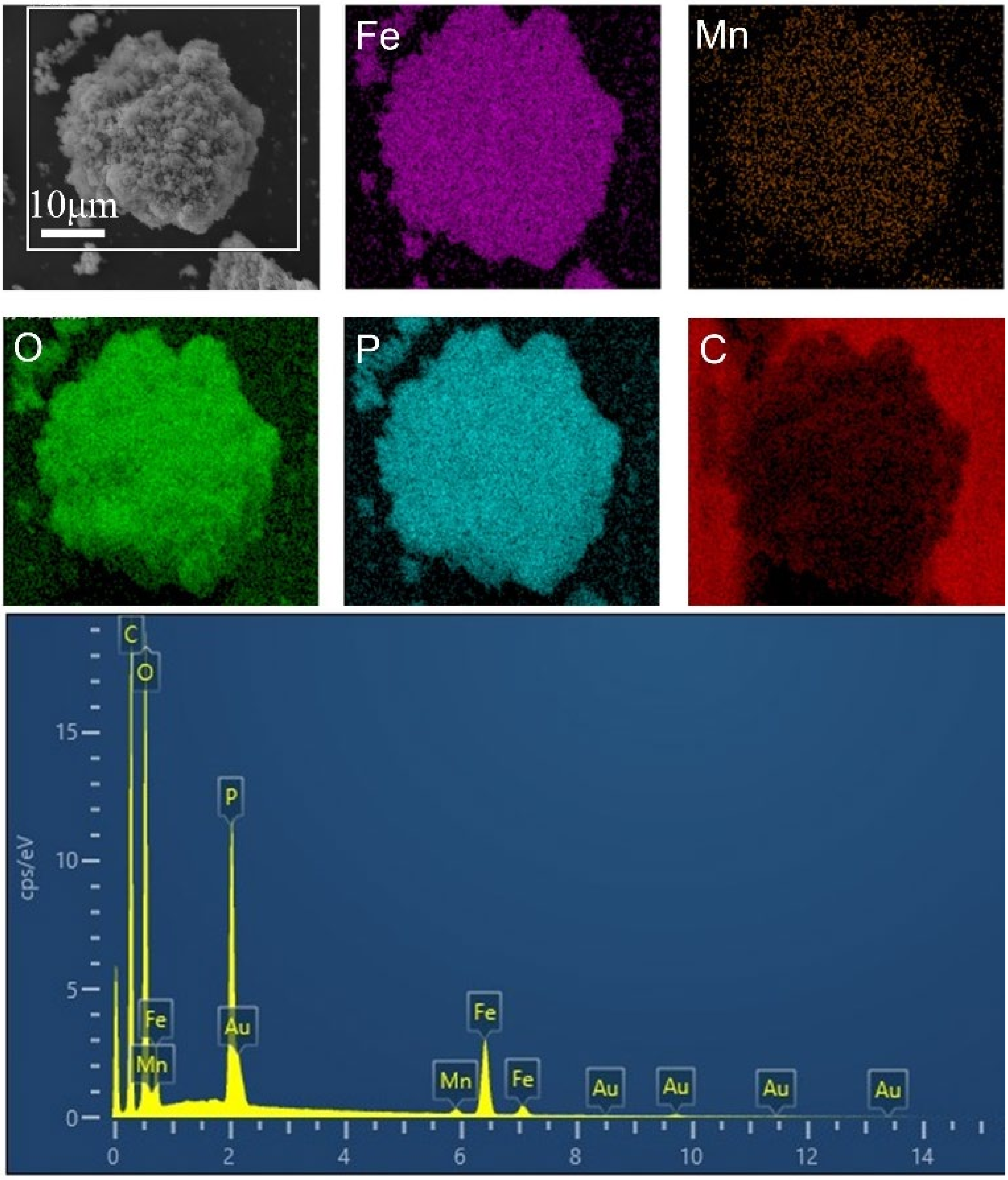

Figure S7. The SEM image and the corresponding elemental mapping images for the selected area of LFP/C−Mn_3 particle.

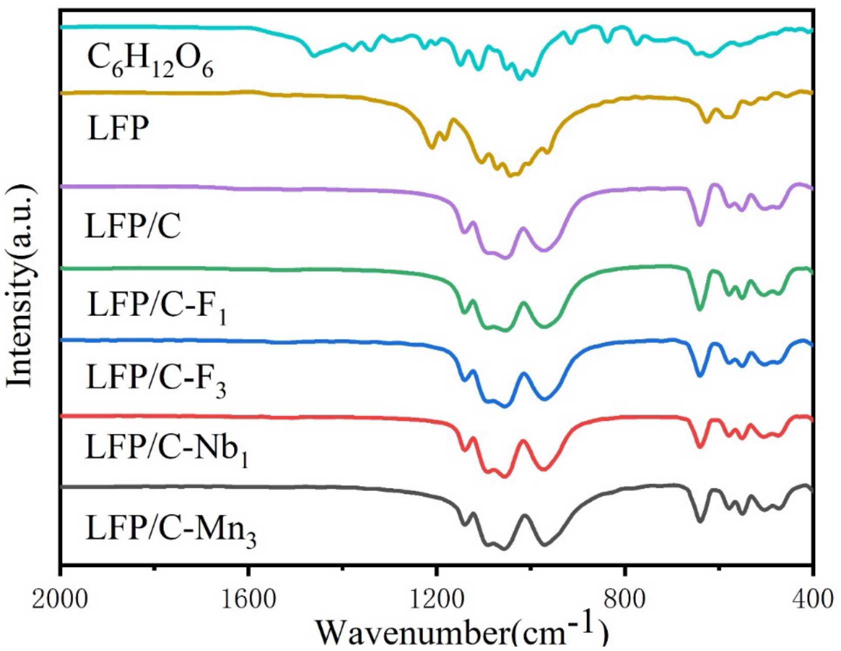

Figure S8. FT−IR spectra of $C_6H_{12}O_6$, LFP, LFP/C and LFP/C−X_n cathode materials.

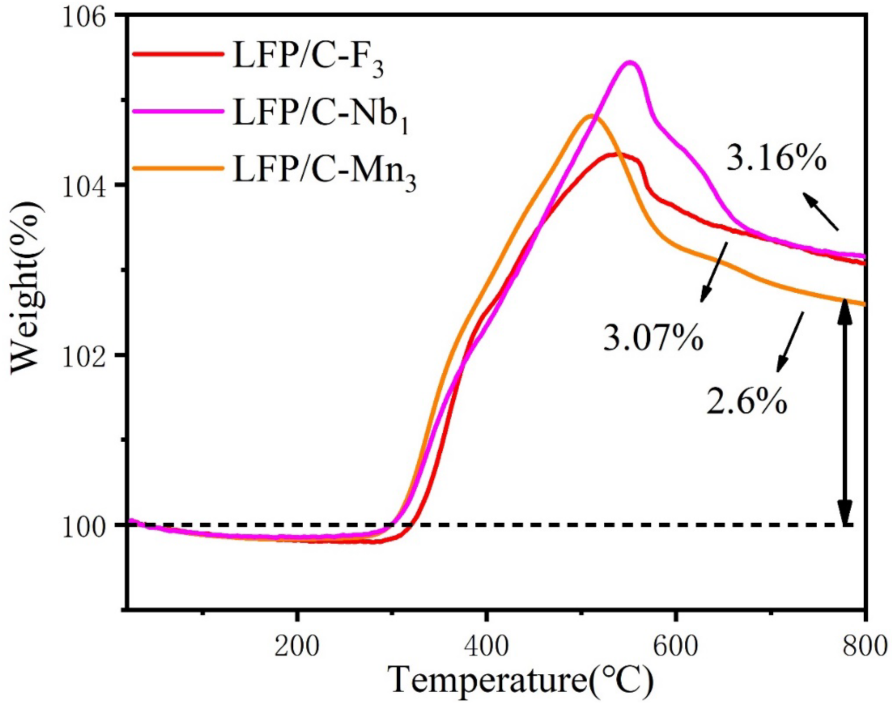

Figure S9. TGA curves of LFP/C−Nb_1, LFP/C−F_3, LFP/C−Mn_3.

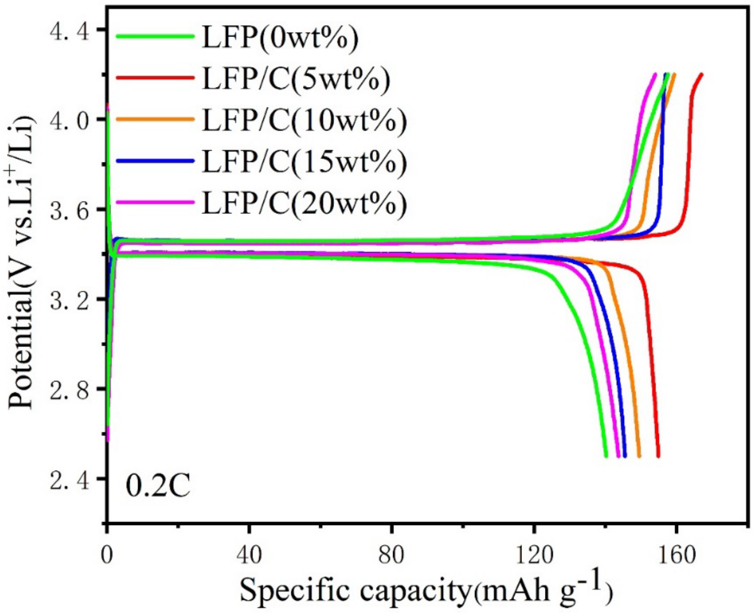

Figure S10. Charge−discharge curves of LFP/C samples with different glucose contents.

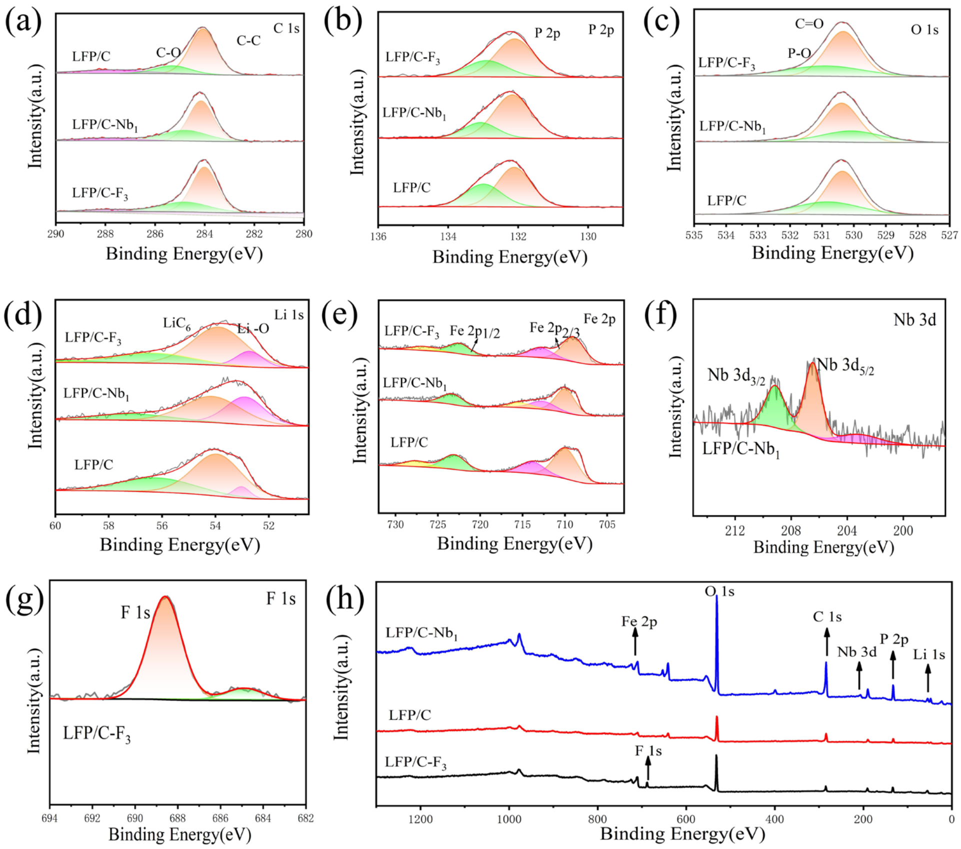

Figure S11. XPS spectra and the fitting results of LFP/C−F_3, LFP/C−Nb_1 and LFP/C for cathode materials. (a) C 1s spectra, (b) P 2p spectra, (c) O 1s spectra, (d) Li 1s spectra, (e) Fe 2p spectra, (f) Nb 3d spectra, (g) F 1s spectra and (h) the full spectrum of the as−prepared samples.

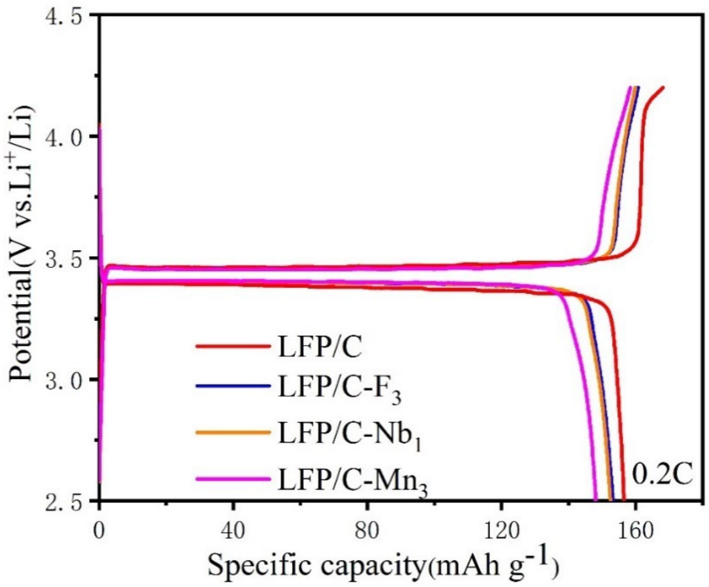

Figure S12. The first−cycle charge−discharge curves of LFP/C and LFP/C−X_n cathodes.

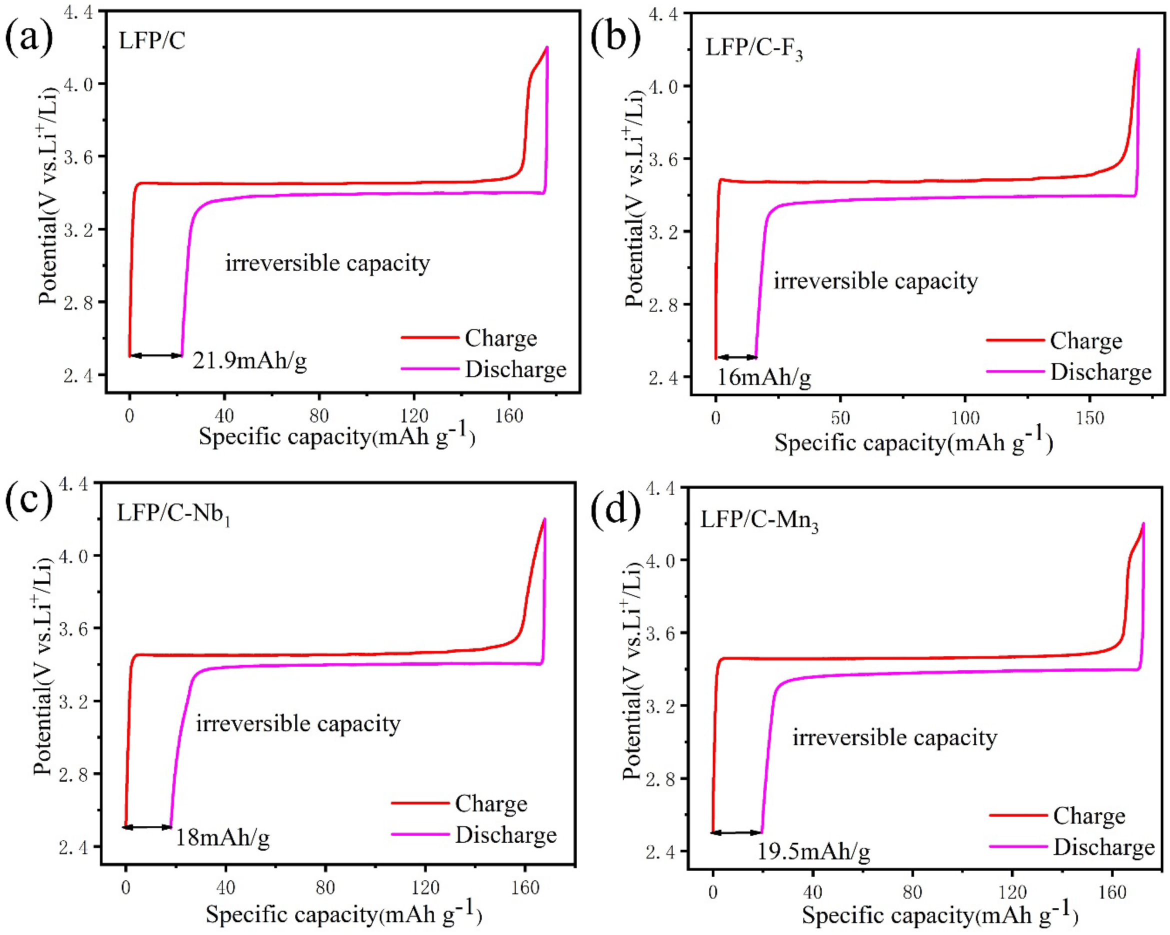

Figure S13. The first charge−discharge curves of the (a) LFP/C, (b) LFP/C−F_3, (c) LFP/C−Nb_1, and (d) LFP/C−Mn_3 cathodes at 0.2 C.

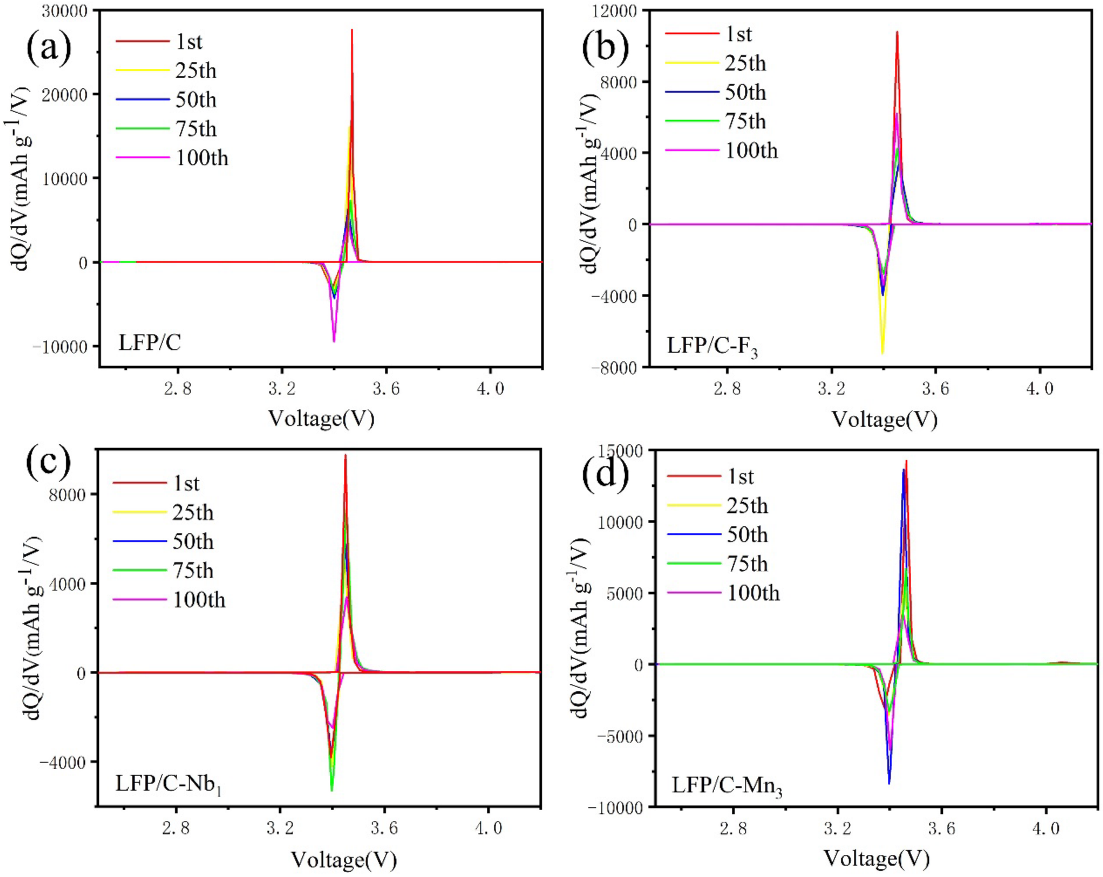

Figure S14. The selected dQ/dV curves of (a) LFP/C, (b) LFP/C−F_3, (c) LFP/C−Nb_1 and (d) LFP/C−Mn_3 at 0.2C in different cycles.

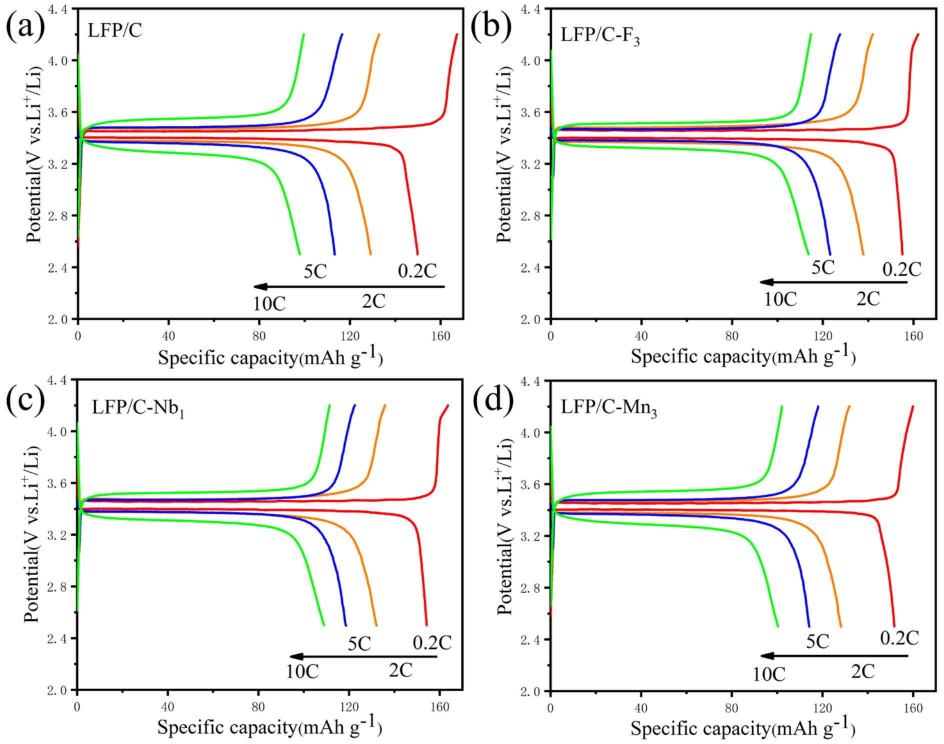

Figure S15. Charge−discharge curves of (a) LFP/C, (b) LFP/C−F_3, (c) LFP/C−Nb_1 and (d) LFP/C−Mn_3 under different charge/discharge rates.

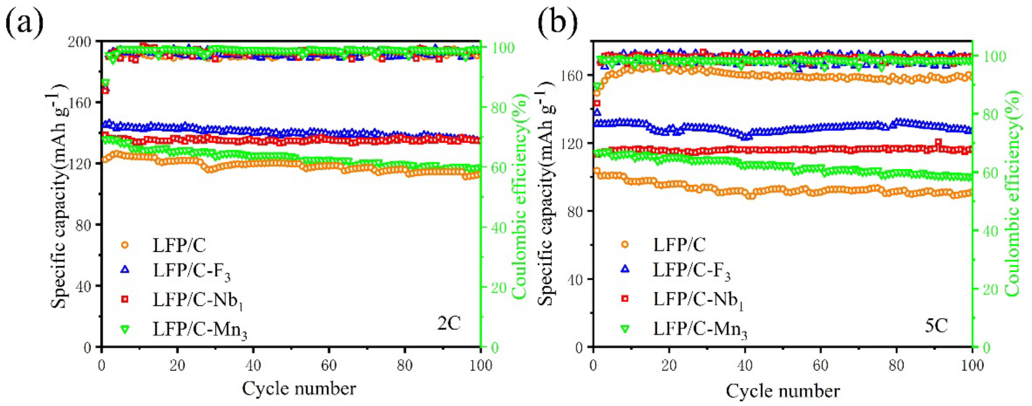

Figure S16. Cycling performance of LFP/C, LFP/C−F_3, LFP/C−Nb_1, and LFP/C−Mn_3 samples at (a) 2 C and (b) 5C in the range of 2.5−4.2 V at 25 °C.

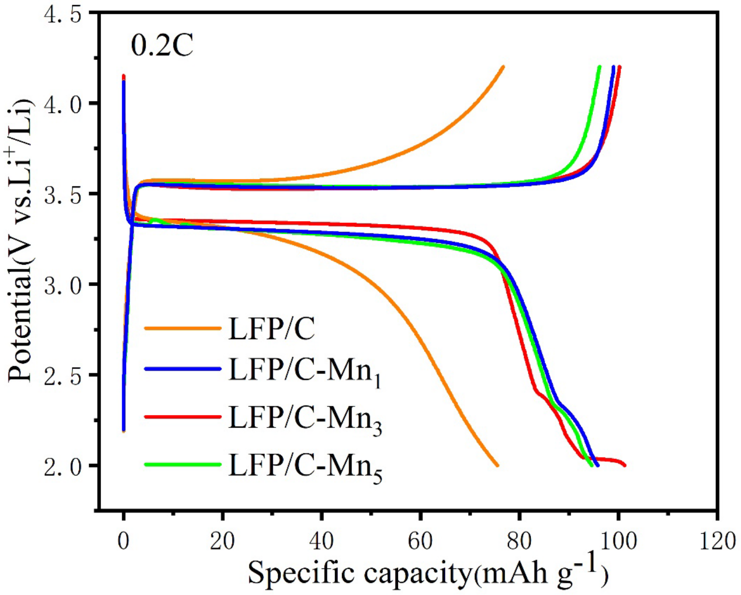

Figure S17. The charge−discharge curves of Mn−doped LFP/C cathodes at 0.2 C at low temperature −15 °C.

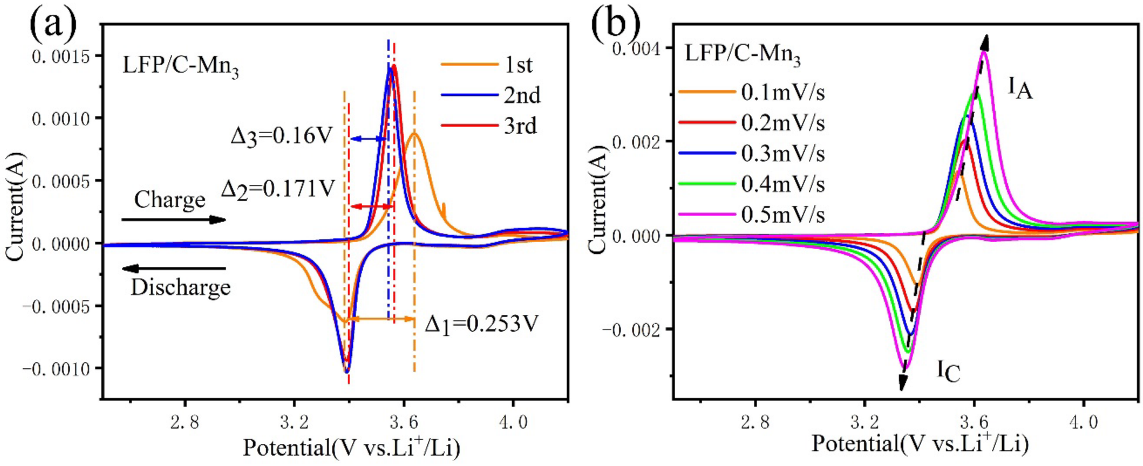

Figure S18. CV curves of (a) LFP/C−Mn3 at a scan rate of 0.1 mV s^{-1} and (b) LFP/C−Mn3 at various scan rates.

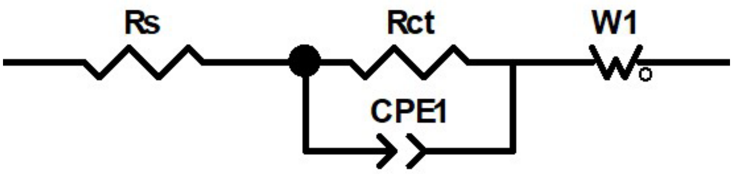

Figure S19. Equivalent circuit diagram fitted and used in EIS data.

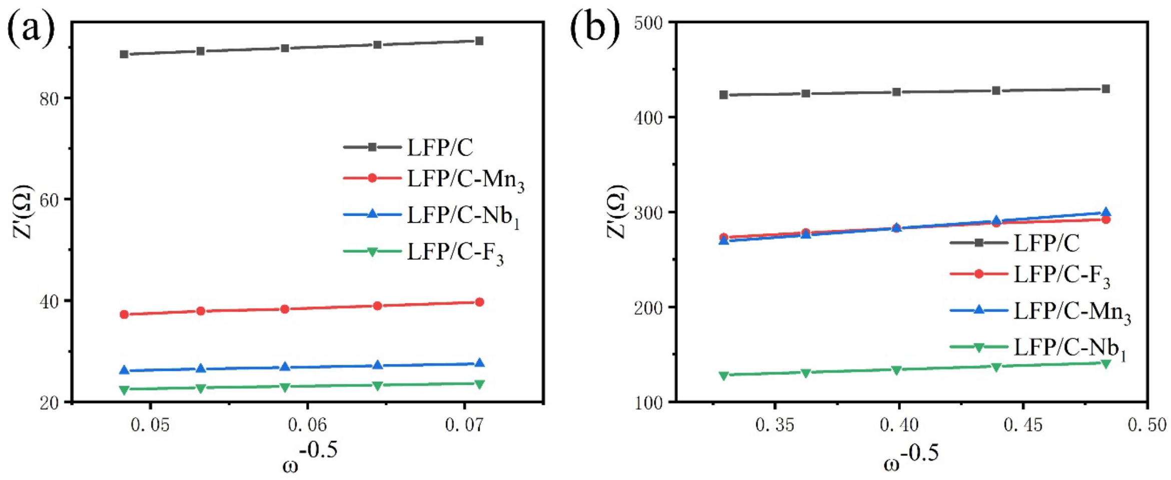

Figure S20. The relationship between low−frequency Z′ and $\omega^{-0.5}$ values derived from EIS results of LFP/C, LFP/C−F_3, LFP/C−Nb_1 and LFP/C−Mn_3 samples after (a) 3 cycles and (b)100 cycles.

Table S1. Structural parameters obtained from Rietveld refinements of XRD patterns of the LFP/C and dopant−modified cathode materials [1,2].

Samples	Lattice Parameter (Å)			Volume (Å^3)	Rwp%	Rp%
	a	*b*	*c*			
LFP/C	10.3244	6.0064	4.6901	290.845	1.564	1.28
LFP/C−F3	10.3190	6.0027	4.6894	290.470	1.508	1.23
LFP/C−Nb1	10.3141	6.0005	4.6891	290.207	1.367	1.12
LFP/C−Mn3	10.3210	6.0048	4.6897	290.647	1.502	1.21

Table S2. Rietveld refinement results of the LFP cathode materials modified by doping.

Sample	Atom	Position	x	y	z
LFP/C	Fe	4c	0.28246	0.25	0.9703
	P	4c	0.0946	0.25	0.4247
	O	4c	0.0937	0.25	0.7489
	O	4c	0.4518	0.25	0.2134
	O	8d	0.1605	0.0544	0.2796
	Li	4a	0	0	0
LFP/C−F3	Fe	4c	0.28234	0.25	0.9709
	P	4c	0.0939	0.25	0.4247
	O	4c	0.094	0.25	0.7472
	O	4c	0.4526	0.25	0.2074
	O	8d	0.1621	0.057	0.2804
	Li	4a	0	0	0
	F	4c	0.094	0.25	0.7472
LFP/C−Nb1	Fe	4c	0.28218	0.25	0.9708
	P	4c	0.094	0.25	0.4268
	O	4c	0.0911	0.25	0.7513
	O	4c	0.4509	0.25	0.2152
	O	8d	0.1615	0.0593	0.2784
	Li	4a	0	0	0
	Nb	4a	0	0	0
LFP/C−Mn3	Fe	4c	0.28221	0.25	0.97472
	P	4c	0.09486	0.25	0.41827
	O	4c	0.09697	0.25	0.7426
	O	4c	0.4572	0.25	0.20584
	O	8d	0.16557	0.04656	0.28492
	Li	4a	0	0	0
	Mn	4c	0.28221	0.25	0.97472

Table S3. Slope values of fitted ip–$v^{1/2}$ curves and diffusion coefficients of charged (D_{OI}) and discharged (D_{RI}) lithium ions of LFP/C, LFP/C−F_3, LFP/C−Nb_1, and LFP/C−Mn_3 cathodes for CV tests at different scan rates.

Sample	I_A slope	I_C slope	D_{OI} ($cm^2\ s^{-1}$)	D_{RI} ($cm^2\ s^{-1}$)
LFP/C	0.00527	−0.00381	4.54×10^{-12}	3.24×10^{-12}
LFP/C−F_3	0.00683	−0.00539	1.49×10^{-11}	1.15×10^{-11}
LFP/C−Nb_1	0.00558	−0.00456	8.53×10^{-12}	7.28×10^{-12}
LFP/C−Mn_3	0.00543	−0.00401	5.16×10^{-12}	4.05×10^{-12}

Table S4. Ohmic resistance (R_s) and charge transfer resistance (R_{ct}) values and calculated Li^+ diffusion diffusion coefficients (D_{Li+}) for LFP/C, LFP/C−F_3, LFP/C−Nb_1, and LFP/C−Mn_3 cathodes after different cycles.

Samples	Cycle	R_s(Ω)	R_{ct}(Ω)	D_{Li+}($cm^2\ s^{-1}$)
LFP/C	3rd	2.681	76.52	2.36×10^{-13}
	100th	5.502	378.50	5.58×10^{-14}
LFP/C−F_3	3rd	1.760	20.63	8.72×10^{-13}
	100th	3.381	199.70	2.15×10^{-13}
LFP/C−Nb_1	3rd	2.509	20.88	7.14×10^{-13}
	100th	3.326	110.10	4.59×10^{-13}
LFP/C−Mn_3	3rd	2.844	38.45	4.06×10^{-13}
	100th	4.231	206.14	1.37×10^{-13}

Table S5. Specific capacity of multiple LFP/C and doped LFP/C samples at different rates.

Samples	0.2C(mAh g^{-1})	2C (mAh g^{-1})	5C (mAh g^{-1})	10C (mAh g^{-1})
LFP/C−1	149.9	129.2	113.3	97.9
LFP/C−2	150.6	129.7	111.8	98.1
LFP/C−3	149.4	127.3	111.5	96.3
LFP/C−F_3−1	155.1	137.9	123.3	113.7
LFP/C−F_3−2	156.3	136.5	122.7	114.2
LFP/C−F_3−3	154.4	136.1	121.3	112.8
LFP/C−Nb_1−1	154.3	132.2	118.7	108.9
LFP/C−Nb_1−2	155.2	133.0	117.2	109.2
LFP/C−Nb_1−3	154.9	131.7	115.5	108.5
LFP/C−Mn_3−1	151.7	128.2	114.2	100.4
LFP/C−Mn_3−2	150.5	129.3	114.7	102.2
LFP/C−Mn_3−3	150.2	129.1	113.6	101.8

Table S6. Standard deviation of LFP/C and doped samples at different rates.

Samples	0.2C	2C	5C	10C
LFP/C	0.602771	1.266228	0.964365	0.986577
LFP/C−F_3	0.960902	0.945163	1.026320	0.709460
LFP/C−Nb_1	0.458258	0.655744	1.601041	0.351188
LFP/C−Mn_3	0.793725	0.585947	0.550757	0.945163

References

1. Jiang, F.; Qu, K.; Wang, M.; Chen, J.; Liu, Y.; Xu, H.; Huang, Y.; Li, J.; Gao, P.; Zheng, J.; Chen, M.; Li, X., Atomic scale insight into the fundamental mechanism of Mn doped $LiFePO_4$. *Sustain. Energy Fuels* **2020,** *4*, 2741-2751.

2. Karimzadeh, S.; Safaei, B.; Huang, W.; Jen, T. C., Theoretical investigation on niobium doped $LiFePO_4$ cathode material for high performance lithium-ion batteries. *J. Energy Storage* **2023,** *67*, 107572.
